# Study on Synergistic Treatment of Pancreatic Cancer by Multiple Small Interfering Ribonucleic Acid Lipid Nanoparticles of Disk Domain Receptor 1, Transforming Growth Factor β1, Tumor-Associated Calcium Signal Transduction Protein 2, and Polyligand Proteoglycan 1

**DOI:** 10.3390/pharmaceutics18070775

**Published:** 2026-06-25

**Authors:** Rongrong Wang, Yiying Zeng, Zhaowu Zeng, Tian Xie

**Affiliations:** 1 School of Pharmacy, Hangzhou Normal University, Hangzhou 311121, China; 19858109512@163.com (R.W.); zengyiying@hznu.edu.cn (Y.Z.); 2 Zhejiang Provincial Key Laboratory of Anti-Cancer Chinese Medicines and Natural Medicines, Hangzhou 311121, China

**Keywords:** disk domain receptor 1, transforming growth factor β1, tumor-associated calcium signal transduction protein 2, polyligand proteoglycan 1, small interfering ribonucleic acid, lipid nanoparticles, pancreatic cancer

## Abstract

**Background/Objective**: This study aimed to use multiple disk domain receptor 1 (DDR1), transforming growth factor β1 (TGFβ-1), tumor-associated calcium signal transduction protein 2 (TACSTD2), and polyligand proteoglycan 1 (SDC1) siRNA to treat pancreatic cancer with the goals of high specificity, significant therapeutic efficacy, and relatively low toxicity. **Methods:** (1) A microfluidic method was used to prepare siRNA-LNPs with different formulations. (2) Quantitative PCR (qPCR) and Western blot assays were used to detect the inhibitory effect of different-prescription siRNA-LNP formulations on mRNA and protein expression levels of related genes in PaTu 8988 pancreatic cells. (3) The anti-pancreatic cancer effect of multiple siRNAs combined with LNPs in vivo was evaluated using the BALB/c nude mouse model with subcutaneous pancreatic cancer xenografts. **Results:** (1) Three siRNA-LNP formulations, DMG, CE 1.5, and CE 0.75, were successfully prepared, exhibiting small particle sizes and uniform distribution. (2) qPCR and Western blot results indicated that DDR1, TGFβ-1, TACSTD2, and SDC1 siRNA-LNP significantly inhibited related genes’ mRNA and protein expression in pancreatic cancer PaTu 8988 cells. (3) Efficacy studies in animals indicated that multiple siRNA combined with LNPs in each group exhibited significant antitumor effects on pancreatic cancer tumor-bearing nude mice. The therapeutic efficacy of the combined siRNAs was superior to that of single siRNA treatments, indicating a clear combined effect, especially with three- and four-siRNA combinations. **Conclusions:** The prepared DDR1/TGFβ-1/TACSTD2/SDC1 siRNA-loaded LNP demonstrated a small particle size, high gene inhibition efficiency, and a significant therapeutic effect in treating pancreatic cancer. Its safety is generally acceptable, but attention should be paid to the toxicity caused by LNP excipients, especially cationic lipids.

## 1. Introduction

Pancreatic cancer is a clinically challenging malignancy with a low survival rate after diagnosis. Surgical resection remains the primary treatment option for non-metastatic pancreatic cancer; however, only 10–20% of patients are eligible for surgery. Despite significant advances in pancreatic cancer therapies, the survival rate remains low, even after surgical resection [1]. However, the incidence and mortality rate of pancreatic cancer continues to rise globally.

Small interfering ribonucleic acids (siRNAs) are double-stranded, non-coding RNA molecules that are about 19–25 base pairs long. siRNA binds to target messenger RNA (mRNA), specifically to mRNA through the RNA-mediated silencing complex (RISC; RNA-induced silencing complex) base pairing [2], induces mRNA degradation, and silences the gene coding for the mRNA [3]. Several siRNA drugs approved by the Food and Drug Administration (FDA), such as Patisiran [4], Patisiran (Onpattro™) was approved by the FDA in 2018 for the treatment of polyneuropathy caused by hereditary thyroxin-mediated amyloidosis [5].

Many nanocarrier systems have been developed for siRNA delivery to tumor tissue [6], and lipid nanoparticles (LNPs) have indicated great potential for delivering nucleic acid-based drugs [7,8]. LNPs are vesicles with a lipid core composed primarily of cholesterol, phospholipids [9,10], polyethylene glycol (PEG)-conjugated lipids, and ionizable cationic lipids. PEG chains are located on the nanoparticles’ surface, helping to prevent serum protein adsorption and uptake by the mononuclear phagocytic system, thereby extending the cycle time in vivo [11]. The primary preparation method of siRNA-LNP is the microfluidic method, which can control the particle size and has high siRNA encapsulation efficiency [12,13]. LNPs have low toxicity, excellent siRNA complexation, high transfection efficiency, and good pharmacokinetic properties [14].

Lipid components containing ionizable cations are crucial for siRNA encapsulation and endosomal escape within cells [15]. Cationic liposomes comprise lipids and polymers composed of cationic head groups, lipophilic tail groups, and junction points [16]. The positive charge of cationic lipids is conducive to transmembrane transport [17]. Optimizing the structure of ionizable lipids and the acid dissociation constant significantly improves the potency of LNPs; however, cationic lipids interact with proteins in the blood and exhibit cytotoxicity [18]. Therefore, balancing the ratio of positive and negative ions reduces toxic side effects and improves drug efficacy [19]. Ionizable cationic lipids ensure efficient siRNA encapsulation [20], help maintain a near-neutral surface charge on the LNPs at physiological pH, and promote endosomal escape, leading to a strong gene knockout effect [21]. DLin-MC3-DMA is a cationic lipid that carries a positive charge under acidic conditions, enabling it to effectively encapsulate negatively charged siRNA through electrostatic interaction. For LNPs containing DLin-MC3-DMA, it was identified that the siRNA gene silencing effect was better, and toxicity was lower in mouse animal experimental studies [22].

Disk domain receptor 1 (DDR1) is a transmembrane receptor belonging to the receptor tyrosine kinase family [23,24]. DDR1 is involved in cell differentiation, proliferation, adhesion, migration, and invasion [25]. DDR1 plays a crucial role in treating various cancers by regulating the interaction between tumor cells and the surrounding collagen matrix. DDR1 stimulates immune rejection by promoting the arrangement of collagen fibers. DDR1 is upregulated in pancreatic ductal adenocarcinoma, and inhibiting DDR1 expression can prevent tumor growth [26]. Transforming growth factor β1 (TGFβ-1), a secreted protein induced by TGFβ, is a multifunctional cell growth factor that plays a role in cell proliferation, differentiation, migration, and apoptosis and is involved in tumor angiogenesis and immune system regulation [27]. Silencing the expression of TGFβ in the tumor microenvironment [28] promotes the differentiation of neutrophils toward antitumor phenotypes, and TGFβ modulates neutrophilic polarization, which is a promising therapeutic strategy for pancreatic cancer [29]. TGFβ-1 is an activator of EMT signal; the upregulation of TGFβ-1 promotes EMT and thus accelerates cancer cell metastasis. c-Myc promotes the infinite proliferation of pancreatic cancer cells, and TGFβ-1 activates transcription factor c-Myc to induce RAP2 expression, resulting in the improved invasiveness of pancreatic cancer cells. TGFβ-1 can enhance the expression of RAP2 at both the mRNA and protein levels, and the inhibition of TGFβ-1 expression can inhibit the invasion of pancreatic cancer cells [30]. Tumor-associated calcium signal transduction protein 2 (TACSTD2) is composed of 323 amino acids [31,32]. TACSTD2 can promote tumor cell proliferation, regeneration, migration, and invasion. The study found that 109 out of 197 patients with pancreatic cancer overexpressed TACSTD2 [33], and TACSTD2 overexpression promoted the growth of tumor cells, indicating that TACSTD2 could be used as a therapeutic target for pancreatic cancer. SDC-1, a heparin sulfate (HS) on the cell surface and extracellular matrix [34], is essential for the maintenance of epithelial phenotype. SDC-1 is considered a membrane-binding protein that binds fibroblast growth factor and vascular endothelial growth factor [35] through the HS chain, thereby stabilizing growth factors. As a surface membrane protein whose cell surface is regulated by KRAS, SDC-1 promotes tumor growth by promoting amino acid metabolism in pancreatic cancer and is a basic metabolic pathway to promote PDAC cell growth. SDC-1 deletion inhibits the growth of subcutaneous xenografts [36,37].

**Figure 1:**
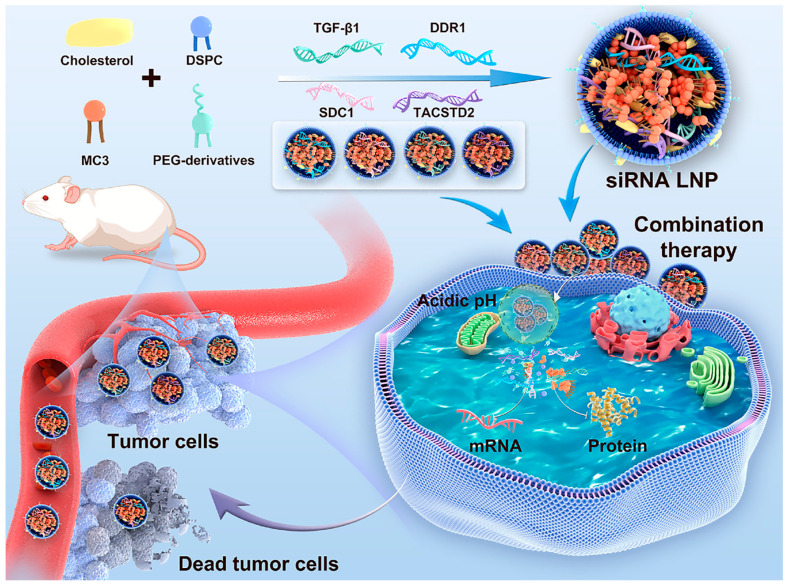
The mechanism of action of siRNA lipid nanoparticles (LNPs).

In this study, PEG CE was proposed as a stabilizer of LNPs as an alternative to PEG_2000_-C-DMG for treating pancreatic cancer. DDR1, TGFβ-1, TACSTD2, and SDC1 genes were used as targets for treating pancreatic cancer. The mechanism of action is shown in Figure 1. Multiple siRNA types of DDR1, TGFβ-1, TACSTD2, and SDC1 were loaded into LNPs for the treatment of pancreatic cancer simultaneously, and their combined effects were observed to enhance efficacy and safety.

## 2. Materials and Methods

INano L rapid nanomedicine preparation system (Maianna Company, Shanghai, China). Spark type micrometer (Tecan, Männedorf, Switzerland). Nicomp Z3000, nanometer particle size Potentiometer (PSS, Palm Springs, CA, USA). Transmission electron microscope (TEM) (Tecnai 12, Philips company, Amsterdam, The Netherlands). UV Photometer (METTler Company, Shanghai, China). General gradient polymerase chain reaction (PCR) instrument (BIO-RAD, Hercules, CA, USA). Real-time fluorescent quantitative PCR instrument (Roche, Basel, Switzerland). Electrophoresis apparatus (BIO-RAD, CA, USA). Trans-Blot Turbo All-Purpose Protein Transfer System (BIO-RAD, CA, USA). Gel Imaging System (BIO-RAD, CA, USA). Enzyme-labeler (Tecan, Männedorf, Switzerland). Laser scanning confocal microscope, FV3000RS (Olympus, Tokyo, Japan). In vivo three-dimensional imaging system for small animals (PerkinElmer, Waltham, MA, USA). Pathologic tissue drying instrument TEC 2500 (Haosiling Instrument Equipment Co., Ltd., Changzhou, China). Rotary microtome RM 2235 (LEICA, Wetzlar, Germany).

DLin-MC3-DMA (AVT Pharmaceutical Technology Co., Ltd., Shanghai, China). DSPC (AVT Pharmaceutical Technology Co., Ltd., Shanghai, China). Cholesterol (AVT Pharmaceutical Technology Co., Ltd., Shanghai, China). PEG2000-C-DMG (AVT Pharmaceutical Technology Co., Ltd., Shanghai, China). PEG5000-cholesterol (CE or PEG CE) was purchased from Shanghai ponsure biotechnology Co., Ltd., China. FastPure® Cell/Tissue Total RNA Isolation Kit V2 Kit (Nuoweizan Biotechnology Co., Ltd., Nanjing, China). HiScript® III All-in-one RT SuperMix Perfect for qPCR Kit (Nuoweizan Biotechnology Co., Ltd., Nanjing, China). SYBR Green Pro Taq HS Premixed qPCR kit (Ecorui Biotechnology Co., Ltd, Changsha, China). RPMI 1640 Medium (Thermo Fisher Scientific, NY, USA). Total Protein Extraction Kit (Jiangsu Kaiji Biotechnology Co., Ltd, Nanjing, China). BCA protein quantitative kit (Nuoweizan Biotechnology Co., Ltd., Nanjing, China). 10% SDS (Coolaber, Beijing, China). Protein Marker (Nuoweizan Biotechnology Co., Ltd., Nanjing, China). NcmECL Ultra Hypersensitive Chemiluminescence Kit (XinSemei Biotechnology Co., Ltd., Suzhou, China). Fetal bovine serum (FBS) (ExCell Bio, Shanghai, China). Cypate (Xinqiao Biological Co., Ltd., Hangzhou, China). Hematoxylin-eosin Dye Solution Kit (Jiangsu Kaiji Biotechnology Co., Ltd., Suzhou, China).

Pancreatic cancer PaTu 8988 cells, Jiangsu Kaigi Biotechnology Co., Ltd, Nanjing, China. Culture conditions: RPMI-1640 culture medium, with 1% penicillin–streptomycin (PS) solution, 10% FBS, placed in 37 °C, 5% CO_2_, and saturated in humidity cell incubator for culture.

### 2.1. siRNA Primer Sequence

DDR1 siRNA: sense (5′-3′): CAAGGCTGAACGGAGGGTGTTG, antisense (5′-3′): GGGCGGTTGTTGATGAGGATAGTG, Sangon Biotech (Shanghai) Co., Ltd., Shanghai, China. TGFβ-1 siRNA: sense (5′-3′): AGCAACAATTCCTGGCGATACCTC, antisense (5′-3′): TCAACCACTGCCGCACAACTC, Sangon Biotech (Shanghai) Co., Ltd., Shanghai, China. TACSTD2 siRNA: sense (5′-3′): CCACCAACAAGATGACCGTG, antisense (5′-3′): CTTGAGCAGCAGACACTTGG, Sangon Biotech (Shanghai) Co., Ltd., Shanghai, China. SDC1 siRNA: sense (5′-3′): CCAAGGAGGGAGAGGCTGTAGTC, antisense (5′-3′): GTGGTGGCTGTGGTCGTTGAG, Sangon Biotech (Shanghai) Co., Ltd., Shanghai, China.

### 2.2. Preparation and Characterization

In this study, siRNA-LNP was prepared using a microfluidic method with a flow rate of 12 mL/min and a ratio of aqueous phase to organic phase of 3:1 as the continuous flow technology. Ethanol, sodium acetate buffer, and free siRNA were removed using a 100 kDa ultrafiltration centrifuge tube. The volume was adjusted using a phosphate buffer, and the final sample was then obtained following sterilization and filtration using a 0.22 μm disposable filter. siRNA-LNP was formulated with a DMG prescription (lipid–nucleic acid ratio 12:1, 6:1), CE 1.5 prescription (lipid–nucleic acid ratio 12:1, 6:1), and CE 0.75 prescription (lipid–nucleic acid ratio 12:1, 6:1) were prepared. The microfluidic technique allowed for precise control over nanoparticles and achieved high siRNA encapsulation efficiency. The particle size and potential of siRNA-LNP were measured using a nanometer particle size potentiometer. The morphology of siRNA-LNP was characterized using TEM. The content of siRNA in siRNA-LNP was detected by the RiboGreen fluorescence method.

**Figure 2:**
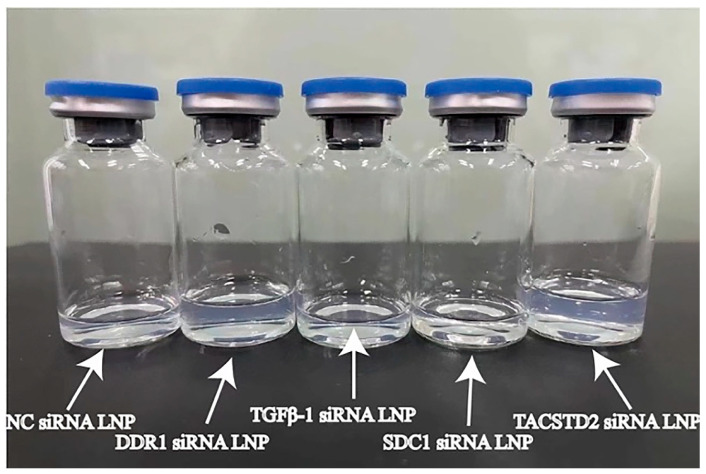
Schematic representation of siRNA-LNPs.

**Figure 3:**
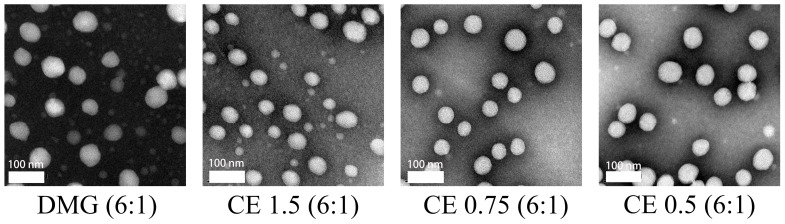
Electron microscope illustration of siRNA-LNPs.

The siRNA-LNP prepared with DMG, CE 1.5, CE 0.75, and CE 0.5 prescriptions was a micro-opalescent transparent liquid in appearance, as depicted in Figure 2. For a total of 97,000 times, TEM was used to observe the LNPs of siRNA with different prescriptions. The siRNA-LNPs with different prescriptions exhibited similar morphologies: they were all round or elliptical, the particle size was about 50–100 nm, the size was slightly different, and the dispersion was relatively uniform, as illustrated in Figure 3. Pictured after prescription optimization, the particle size and particle size distribution of siRNA-LNPs of different genes are depicted in Table 1, with an average particle size of about 80–100 nm, a PDI of about 0.1, 90% < 200 nm, a small particle size, and uniform distribution. The siRNA-LNP prepared by CE prescription was smaller than that prepared by DMG. The PDI was slightly larger compared to that of the DMG prescription. Following prescription optimization, the potential of siRNA-LNPs of different genes is illustrated in Table 2. The positive charge in the 10 mM pH 4 buffer and the negative charge in the 10 mM pH 7.4 buffer are relatively stable, making siRNA-LNP not easily engulfed by the mononuclear macrophage system in blood circulation. It is positively charged in intracellular endosomes or lysosomes and can interact with endosomes or lysosomes, escape from the endosomes or lysosomes, and distribute in the cytoplasm, thereby silencing the mRNA expression of specific genes. The siRNA concentration in the different-gene siRNA-LNP was measured at approximately 250 µg/mL, closely matching the theoretical content. The loss of siRNA during the siRNA enveloping process of LNPs was less, and the prepared siRNA-LNP was relatively stable.

DMG Prescription: MC3/DSPC/cholesterol/PEG2000-C-DMG in a molar ratio of 50/10/38.5/1.5.

CE 1.5 Prescription: MC3/DSPC/cholesterol/PEG CE with the molar ratio 45/10/43.5/1.5.

CE 0.75 Prescription: MC3/DSPC/cholesterol/PEG CE with the molar ratio 45/10/44.25/0.75.

CE 0.5 Prescription: MC3/DSPC/cholesterol/PEG CE with the molar ratio 45/10/44.5/0.5.

DMG (6:1) Prescription: MC3/DSPC/cholesterol/PEG2000-C-DMG in a molar ratio of 50/10/38.5/1.5, lipid–nucleic acid ratio of 6:1.

DMG (12:1) Prescription: The MC3/DSPC/cholesterol/PEG2000-C-DMG molar ratio was 50/10/38.5/1.5, and the lipid–nucleic acid ratio was 12:1.

CE 1.5 (6:1) Prescription: The MC3/DSPC/cholesterol/PEG molar ratio was 45/10/43.5/1.5, and the lipid–nucleic acid ratio was 6:1.

CE 1.5 (12:1) Prescription: The MC3/DSPC/cholesterol/PEG CE molar ratio was 45/10/43.5/1.5, and lipid–nucleic acid ratio was 12:1.

### 2.3. Study on Inhibition of siRNA-LNP Gene Expression

To study the inhibitory effects of DDR1 siRNA-LNP, TGFβ-1 siRNA-LNP, TACSTD2 siRNA-LNP, and SDC1 siRNA-LNP on mRNA, and the protein expression of related genes in pancreatic cancer PaTu 8988 cells, qPCR and Western blot analyses were performed. These techniques were used to detect mRNA and protein expression levels of pancreatic cancer PaTu 8988 cells treated with siRNA-LNP formations of various prescriptions. The results aimed to evaluate the effectiveness of different siRNA-LNP formulations on mRNA and the protein expression of pancreatic cancer PaTu 8988 cells. See the Supplementary Materials for the results of mRNA expression levels.

The specific steps of the qPCR method are described in the Supplementary Materials.

The specific steps of the Western blot assay are described in the Supplementary Materials.

**Figure 4:**
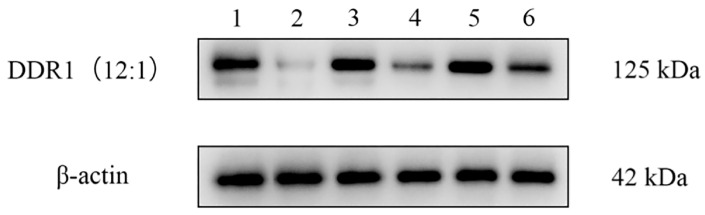
Protein expression bands of pancreatic cancer PaTu 8988 cells treated with different prescriptions of DDR1 siRNA-LNP (lipid–nucleic acid ratio of 12:1) (*n* = 4). 1: DMG NC siRNA-LNP (12:1), 2: DMG DDR1 siRNA-LNP (12:1), 3: CE 1.5 NC siRNA-LNP (12:1), 4: CE 1.5 DDR1 siRNA-LNP (12:1), 5: CE 0.75 NC siRNA-LNP (12:1), and 6: CE 0.75 DDR1 siRNA-LNP (12:1).

The protein bands of pancreatic cancer PaTu 8988 cells treated with different-prescription DDR1 siRNA-LNPs with the lipid–nucleic acid ratio (12:1) are illustrated in Figure 4.

**Figure 5:**
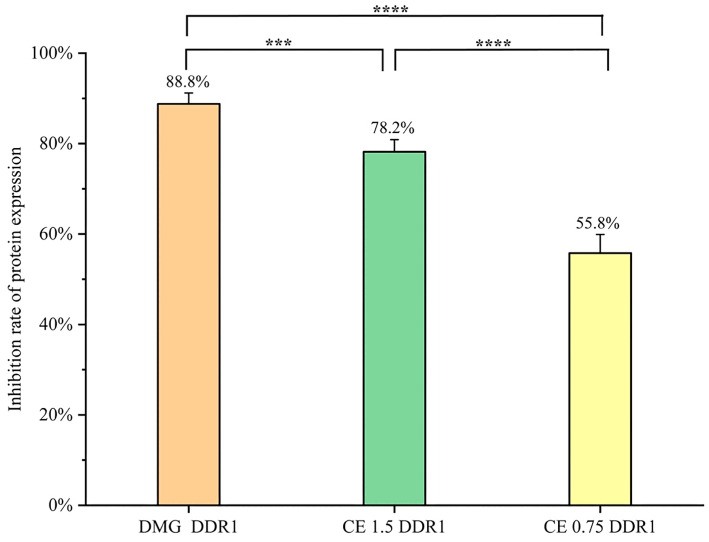
Comparison of the inhibition rate of protein expression in pancreatic cancer PaTu 8988 cells treated with different-prescription DDR1 siRNA-LNPs (lipid–nucleic acid ratio of 12:1). **** *p* < 0.0001; *** *p* < 0.001 (*n* = 4).

Compared with negative control (NC) siRNA-LNP, the protein expression levels in PaTu 8988 pancreatic cancer cells treated with different prescriptions of DDR1 siRNA-LNP formulation were significantly decreased, and the differences were statistically significant. The inhibition rates of protein expression in pancreatic cancer PaTu 8988 cells treated with different DDR1 siRNA-LNP prescriptions were compared, as depicted in Figure 5.

Figure 5 indicates that different formulations of DDR1 siRNA-LNP could effectively inhibit the protein expression of pancreatic cancer PaTu 8988 cells, and DMG DDR1 siRNA-LNP had the highest inhibitory rate of protein expression. The inhibitory effect of CE 1.5 DDR1 siRNA-LNP and CE 0.75 DDR1 siRNA-LNP was weaker than that of DMG DDR1 siRNA-LNP.

**Figure 6:**
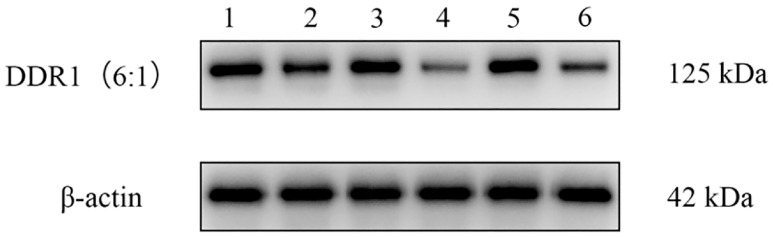
Protein expression bands of pancreatic cancer PaTu 8988 cells treated with different prescriptions of DDR1 siRNA-LNP (lipid–nucleic acid ratio 6:1) (*n* = 4). 1: DMG NC siRNA-LNP (6:1), 2: DMG DDR1 siRNA-LNP (6:1), 3: CE 1.5NC siRNA-LNP (6:1), 4: CE 1.5DDR1 siRNA-LNP (6:1), 5: CE 0.75 NC siRNA-LNP (6:1), 6: CE 0.75 DDR1 siRNA-LNP (6:1).

The protein bands of pancreatic cancer PaTu 8988 cells treated with different-prescription DDR1 siRNA-LNPs with lipid–nucleic acid ratio (6:1), as illustrated in Figure 6.

**Figure 7:**
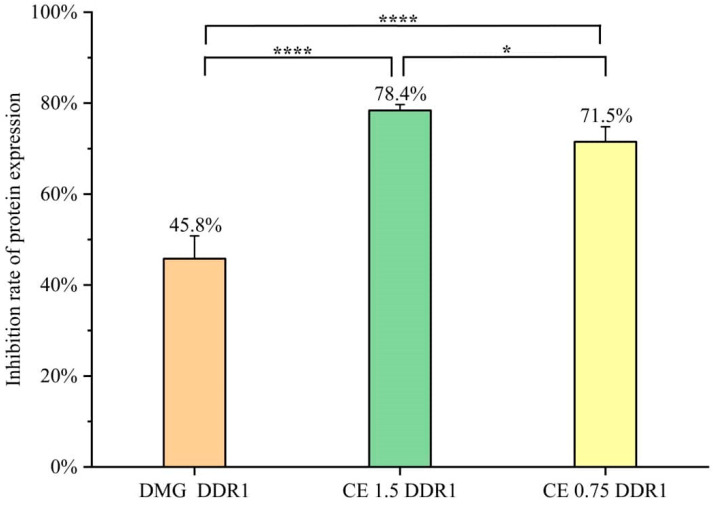
Comparison of the protein expression inhibition rate of pancreatic cancer PaTu 8988 cells treated with different prescriptions of DDR1 siRNA-LNP (lipid–nucleic acid ratio 6:1). **** *p* < 0.0001; * *p* < 0.05 (*n* = 4).

Compared with the LNP of NC siRNA, the protein expression of pancreatic cancer PaTu 8988 cells treated with different prescriptions of DDR1 siRNA-LNP was significantly decreased, and the comparison difference was statistically significant. The inhibition rates of protein expression in pancreatic cancer PaTu 8988 cells treated with different DDR1 siRNA-LNP prescriptions were compared, as indicated in Figure 7.

As illustrated in Figure 7, DDR1 siRNA-LNP with different prescriptions can effectively inhibit the protein expression of pancreatic cancer PaTu 8988 cells. The CE 1.5 DDR1 siRNA-LNP has the highest protein expression inhibition rate. The inhibitory effect of CE 1.5 DDR1 siRNA-LNP and CE 0.75 DDR1 siRNA-LNP was stronger than that of DMG DDR1 siRNA-LNP.

**Figure 8:**
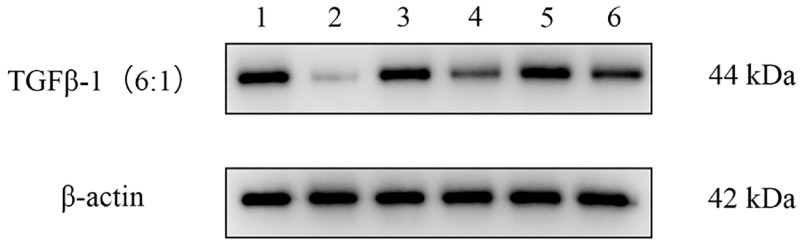
Protein expression bands of pancreatic cancer PaTu 8988 cells treated with different prescriptions of TGFβ-1 siRNA-LNP (lipid–nucleic acid ratio 6:1) (*n* = 4). 1: DMG NC siRNA-LNP (6:1), 2: DMG TGFβ-1 siRNA-LNP (6:1), 3: CE 1.5 NC siRNA-LNP (6:1), 4: CE 1.5 TGFβ-1 siRNA-LNP (6:1), 5: CE 0.75 NC siRNA-LNP (6:1), and 6: CE 0.75 TGFβ-1 siRNA-LNP (6:1).

Protein bands of pancreatic cancer PaTu 8988 cells treated with different-prescription lipid–nucleic acid ratios (6:1) TGFβ-1 siRNA-LNP are displayed in Figure 8.

**Figure 9:**
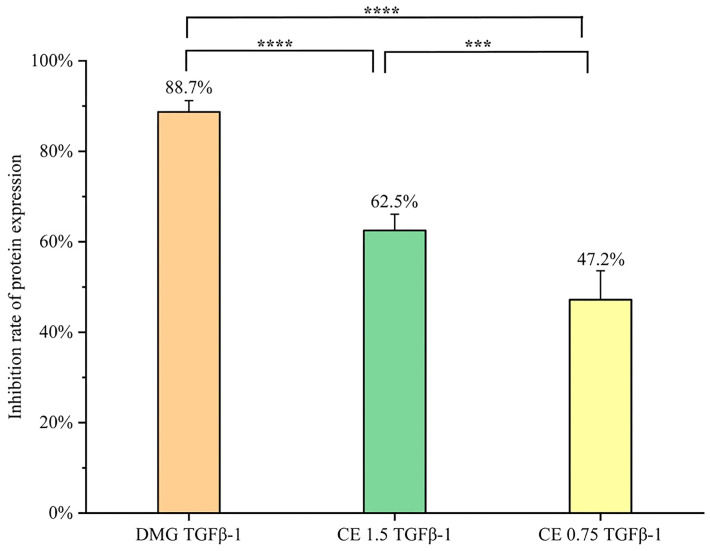
Comparison of the protein expression inhibition rate of pancreatic cancer PaTu 8988 cells treated with TGFβ-1 siRNA-LNP with different prescriptions (lipid–nucleic acid ratio 6:1). **** *p* < 0.0001; *** *p* < 0.001 (*n* = 4).

Compared with the LNP of NC siRNA, the protein expression of pancreatic cancer PaTu 8988 cells treated with different prescriptions of TGFβ-1 siRNA-LNP was significantly decreased, and the differences were statistically significant. The inhibition rates of protein expression in pancreatic cancer PaTu 8988 cells treated with varying prescriptions of TGFβ-1 siRNA-LNP were compared, as depicted in Figure 9.

According to the findings of Figure 9, TGFβ-1 siRNA-LNP with different prescriptions could effectively inhibit the protein expression of pancreatic cancer PaTu 8988 cells, and the inhibition rate of protein expression of DMG TGFβ-1 siRNA-LNP was the highest. The inhibitory effect of CE 1.5 TGFβ-1 siRNA-LNP and CE 0.75 TGFβ-1 siRNA-LNP was weaker than that of DMG TGFβ-1 siRNA-LNP.

**Figure 10:**
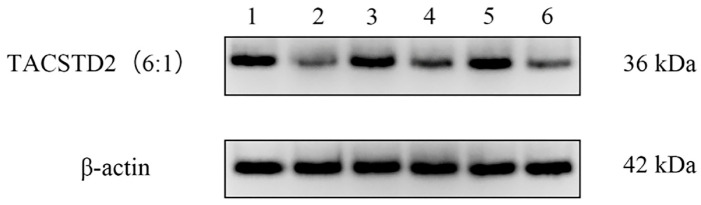
Protein expression bands of pancreatic cancer PaTu 8988 cells treated with different TACSTD2 siRNA-LNP prescriptions (lipid–nucleic acid ratio 6:1) (*n* = 4). 1: DMG NC siRNA-LNP (6:1), 2: DMG TACSTD2 siRNA-LNP (6:1), 3: CE 1.5NC siRNA-LNP (6:1), 4: CE 1.5TACSTD2 siRNA-LNP (6:1), 5: CE 0.75 NC siRNA-LNP (6:1), and 6: CE 0.75 TACSTD2 siRNA-LNP (6:1).

The protein bands of pancreatic cancer PaTu 8988 cells treated with different TACSTD2 siRNA-LNP prescriptions with the lipid–nucleic acid ratio (6:1) are illustrated in Figure 10.

**Figure 11:**
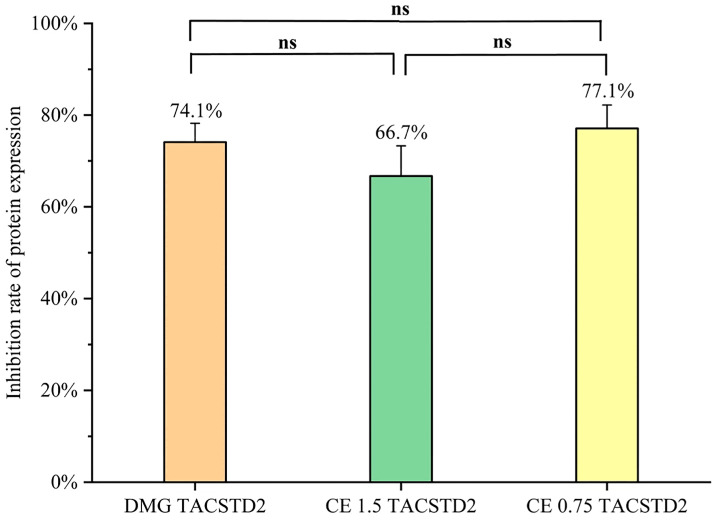
Comparison of protein expression inhibition rate of pancreatic cancer PaTu 8988 cells treated with TACSTD2 siRNA-LNP with different prescriptions (lipid–nucleic acid ratio 6:1). ns means *p* > 0.05 (*n* = 4).

Compared with the LNP of NC siRNA, the protein expression of pancreatic cancer PaTu 8988 cells treated with different TACSTD2 siRNA-LNP prescriptions was significantly decreased, and the differences were highly statistically significant. The inhibition rates of protein expression in pancreatic cancer PaTu 8988 cells treated with TACSTD2 siRNA-LNP with different prescriptions were compared, as illustrated in Figure 11.

As depicted in Figure 11, TACSTD2 siRNA-LNP with different prescriptions can effectively inhibit the protein expression of pancreatic cancer PaTu 8988 cells. CE 1.5 TACSTD2 siRNA-LNP and CE 0.75 TACSTD2 siRNA-LNP exhibited the same inhibitory effect as DMG TACSTD2 siRNA-LNP.

**Figure 12:**
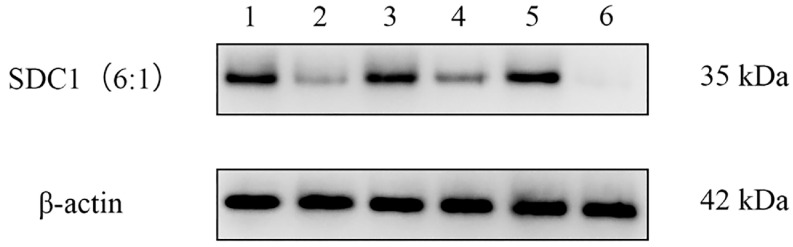
Protein expression bands of pancreatic cancer PaTu 8988 cells treated with different-formulae SDC1 siRNA-LNPs (lipid–nucleic acid ratio 6:1) (*n* = 4). 1: DMG NC siRNA-LNP (6:1), 2: DMG SDC1 siRNA-LNP (6:1), 3: CE 1.5NC siRNA-LNP (6:1), 4: CE 1.5SDC1 siRNA-LNP (6:1), 5: CE 0.75 NC siRNA-LNP (6:1), and 6: CE 0.75 SDC1 siRNA-LNP (6:1).

The protein bands of pancreatic cancer PaTu 8988 cells treated with different-prescription lipid–nucleic acid ratios (6:1) and SDC1 siRNA-LNP are illustrated in Figure 12.

**Figure 13:**
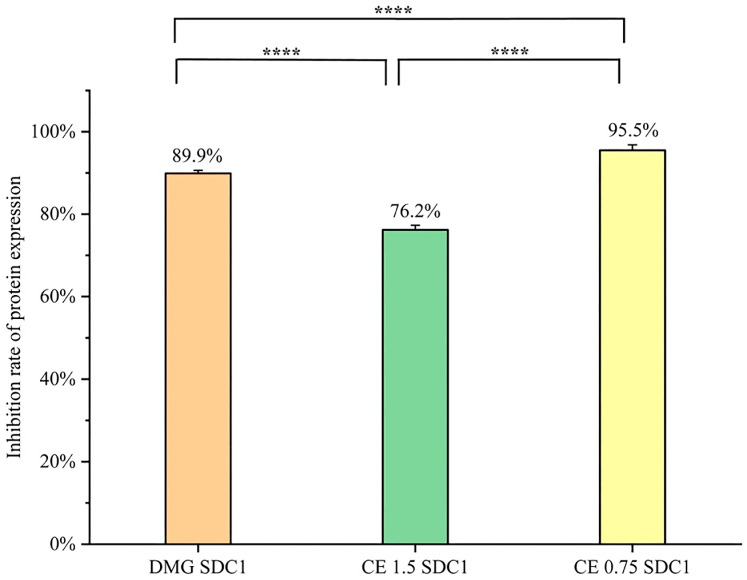
Comparison of the inhibition rate of protein expression in pancreatic cancer PaTu 8988 cells treated with different SDC1 siRNA-LNP prescriptions (lipid–nucleic acid ratio 6:1). **** *p* < 0.0001 (*n* = 4).

Compared with the LNP of NC siRNA, the protein expression of pancreatic cancer PaTu 8988 cells treated with different prescriptions of SDC1 siRNA-LNP was significantly decreased, and the comparative differences were highly statistically significant. The inhibition rates of protein expression in pancreatic cancer PaTu 8988 cells treated with SDC1 siRNA-LNP of different prescriptions were compared, as depicted in Figure 13.

As demonstrated in Figure 13, SDC1 siRNA-LNP with varying prescriptions can effectively inhibit the protein expression of pancreatic cancer PaTu 8988 cells, and the protein expression inhibition rate of CE 0.75 SDC1 siRNA-LNP is the highest. The inhibitory effect of DMG SDC1 siRNA-LNP was stronger compared to that of CE 1.5 SDC1 siRNA-LNP.

The above results indicated that different-gene siRNA-LNPs with different prescriptions had a high inhibitory effect on the protein expression of pancreatic cancer PaTu 8988 cells, and the inhibitory efficiency of various genes and different-prescription siRNA-LNPs on the protein expression of pancreatic cancer PaTu 8988 cells was not wholly consistent. Generally, siRNA-LNPs with DMG prescription, CE 1.5 prescription, and CE 0.75 prescription had excellent inhibitory effects on pancreatic cancer PaTu 8988 cells.

### 2.4. Cell Uptake of siRNA-LNP In Vitro

To study the uptake efficiency of LNPs by pancreatic cancer PaTu 8988 cells, DDR1 siRNA-LNPs with different formulations and different lipid–nucleic acid ratios were labeled with 5′ FAM fluorescent dye and analyzed using scanning confocal microscopy. The intracellular uptake efficiency was assessed by comparing fluorescent intensities in PaTu 8988 cells treated with different fluorescently labeled DDR1 siRNA-LNP formulations and lipid–nucleic acid ratios.

**Figure 14:**
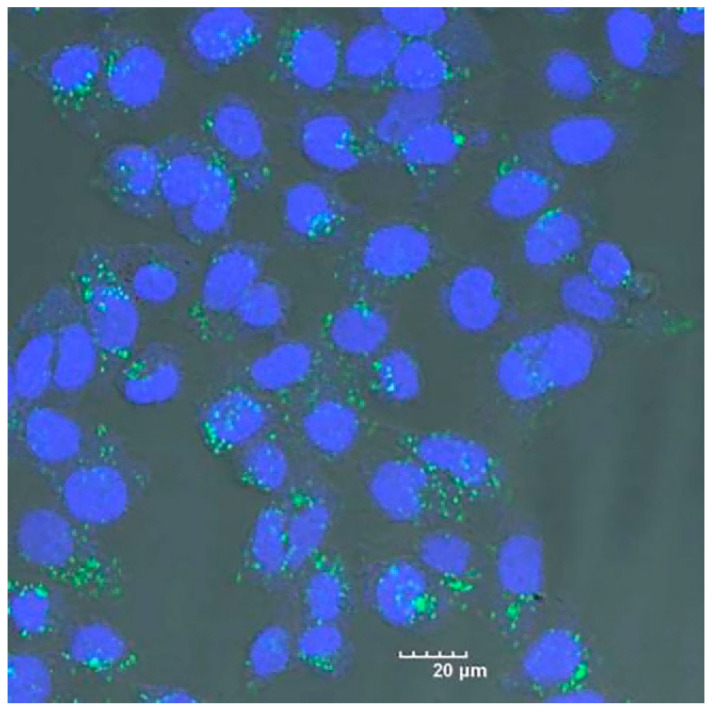
Uptake of 5′ FAM CE 0.75 DDR1 siRNA-LNP (12:1) in pancreatic cancer PaTu 8988 cells at 6 h with the scale of 20 μm. The cell nucleus was indicated with blue fluorescence and the 5′ FAM DDR1 siRNA-LNP demonstrated green fluorescence and distributed in the cell (*n* = 3).

The results indicated that free 5′-FAM-labeled DDR1 siRNA could not enter the cells. In contrast, 5′-FAM-labeled DMG DDR1 siRNA-LNP, 5′ FAM CE 1.5 DDR1 siRNA-LNP, and 5′ FAM CE 0.75 DDR1 siRNA-LNP with lipid–nucleic acid ratio (6:1) and (12:1) could enter cells. The cell outline was observed under a laser-scanning confocal microscope. The cell nucleus was indicated with blue fluorescence due to the DAPI staining solution, and the 5′ FAM DDR1 siRNA-LNP demonstrated green fluorescence and distributed in the cell, as depicted in Figure 14.

**Figure 15:**
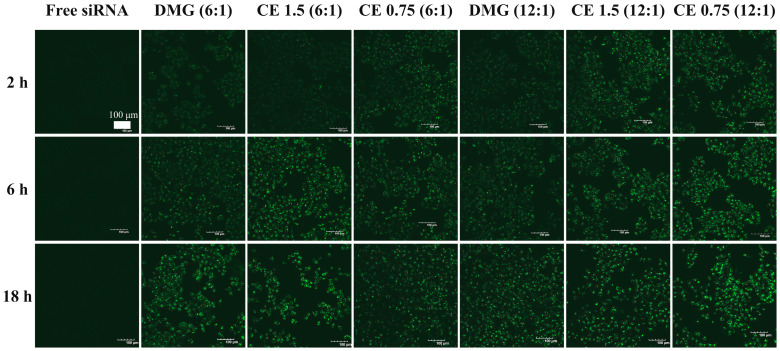
Uptake of 5′ FAM DDR1 siRNA-LNPs with different prescriptions and different lipid–nucleic acid ratios in pancreatic cancer PaTu 8988 cells; 100 μm (*n* = 3).

5′ FAM DDR1 siRNA-LNP indicated green fluorescence and was distributed in cells. The 5′ FAM DMG DDR1 siRNA-LNP, 5′ FAM CE 1.5 DDR1 siRNA-LNP, and 5′ FAM CE 0.75 DDR1 siRNA-LNP with lipid-to-nucleic acid ratios of 6:1 and 12:1, respectively, were detected for pancreatic cancer PaTu. The uptake results of 8988 cells are depicted in Figure 15.

**Figure 16:**
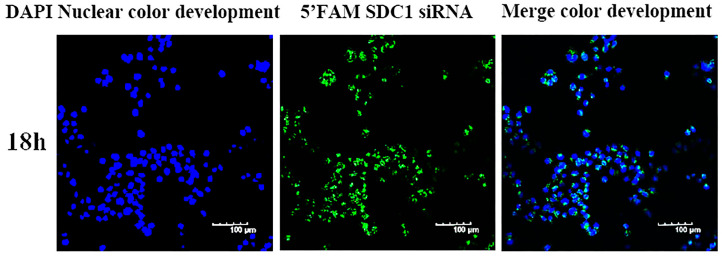
Uptake of 5′ FAM DMG SDC1 siRNA-LNP (6:1) in pancreatic cancer PaTu 8988 cells; scale 100 μm (*n* = 3).

The results indicated that with the prolongation of time, different prescriptions and lipid–nucleic acid ratios of 5′ FAM DDR1 siRNA-LNP gradually entered the cells, and the cell uptake tended to be similar. The cell nucleus was indicated by blue fluorescence due to the DAPI staining solution, and the 5′ FAM DMG SDC1 siRNA-LNP (6:1) demonstrated green fluorescence and dispersed in the cells. Free 5′ FAM DMG SDC1 siRNA-LNP (6:1) failed to enter cells, while 5′ FAM DMG SDC1 siRNA-LNP (6:1) could efficiently enter pancreatic cancer PaTu 8988 cells, as illustrated in Figure 16, indicating that the prepared LNP could carry various siRNA into the cells.

### 2.5. The Tumor Tissue Fluorescence Intensity Dynamics of Cypate in Cypate siRNA LNP

The siRNA-LNPs with different prescriptions and lipid–nucleic acid ratios were labeled with Cypate fluorescence to study the fluorescence intensity changes in siRNA-LNP with Cypate fluorescence labeling in nude mice.

**Figure 17:**
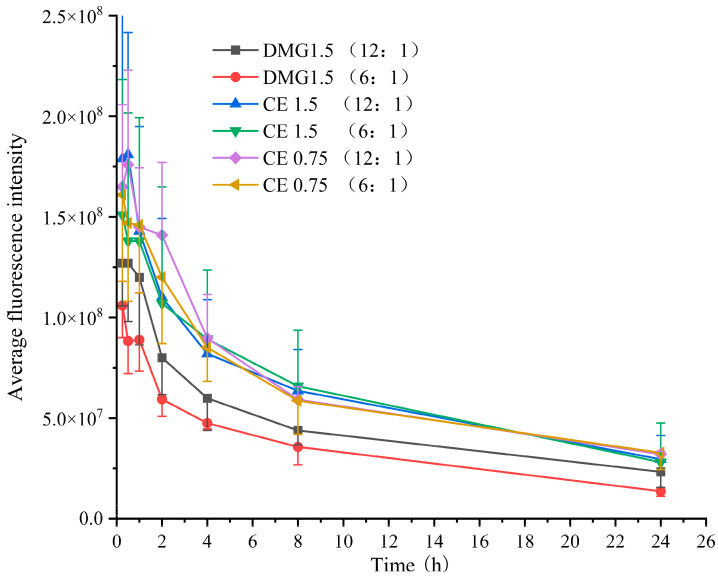
Tumor fluorescence intensity–time curves of Cypate in siRNA-LNPs with different formulations and different lipid/nucleic acid ratios (*n* = 5).

The tumor fluorescence intensity–time curves of Cypate in siRNA-LNPs with different formulations and lipid–nucleic acid ratios are depicted in Figure 17, and the fluorescence intensity gradually decreased with the extension of time. The area under the curve (AUC) and Cmax of DMG siRNA-LNP (12:1) were slightly higher compared to those of DMG siRNA-LNP (6:1), indicating that the ratio of DLin-MC3-DMA to siRNA exhibited a significant effect on the pharmacokinetic parameters for DMG prescription. The AUC (0–t) and Cmax of CE 1.5 siRNA-LNP (12:1) and CE 1.5 siRNA-LNP (6:1) were similar. The AUC (0–t) and Cmax of CE 0.75 siRNA-LNP (12:1) and CE 0.75 siRNA-LNP (6:1) were identical, indicating that the ratio of DLin-MC3-DMA to siRNA had a non-significant effect on the pharmacokinetic parameters for CE prescription. Excellent pharmacokinetic parameters were obtained within this range. The AUC and Cmax of CE 1.5 siRNA-LNP (6:1) and CE 0.75 siRNA-LNP (6:1) were similar. The AUC and Cmax of CE 1.5 siRNA-LNP (6:1) were 1.82 and 1.47 times higher than those of DMG siRNA-LNP (6:1), respectively. The AUC and Cmax of CE 1.5 siRNA-LNP (6:1) were increased by 82% and 47% compared with DMG siRNA-LNP (6:1). The AUC and Cmax of CE 0.75 siRNA-LNP (6:1) were 1.8 and 1.57 times higher than those of DMG siRNA-LNP (6:1), respectively. The AUC and Cmax of CE 0.75 siRNA-LNP (6:1) were increased by 80% and 57% compared with DMG siRNA-LNP (6:1).

These findings suggested that the exposure of CE 1.5 siRNA-LNP (6:1) and CE 0.75 siRNA-LNP (6:1) in tumor tissue was higher than that of DMG siRNA-LNP (6:1). The kinetic parameters of CE 1.5 siRNA-LNP in the tumor were better than those of DMG siRNA-LNP (6:1). Considering that the drug is more complex in human blood than in nude mice and has a more substantial opsonization effect on siRNA-LNP, the choice of CE 1.5 siRNA-LNP (6:1) may be more suitable for treating malignant tumors, including pancreatic cancer.

### 2.6. Pharmacodynamic Study of siRNA-LNP In Vivo

The PaTu 8988 pancreatic cancer cells were used to construct a BALB/c nude mouse model of a subcutaneous transplantation tumor of pancreatic cancer. The therapeutic effect of various gene-targeting siRNA-LNPs was evaluated by tail vein injection. The standard dose was 1 mg/kg, except for the DMG DDR1 siRNA-LNP (6:1) administered at 2 mg/kg. The main research contents included the following: (1) the pharmacodynamic comparison of individual siRNA-LNPs, (2) the pharmacodynamic comparison of different formulations and doses of DDR1 siRNA-LNPs, (3) the pharmacodynamic evaluation of dual siRNA combination delivered through LNPs, and (4) the pharmacodynamic evaluation of multiple siRNA combinations delivered through LNPs.

### 2.7. Inhibition Rate of Pancreatic Cancer Tumor-Bearing Nude Mice in Different siRNA-LNP Administration Groups

**Figure 18:**
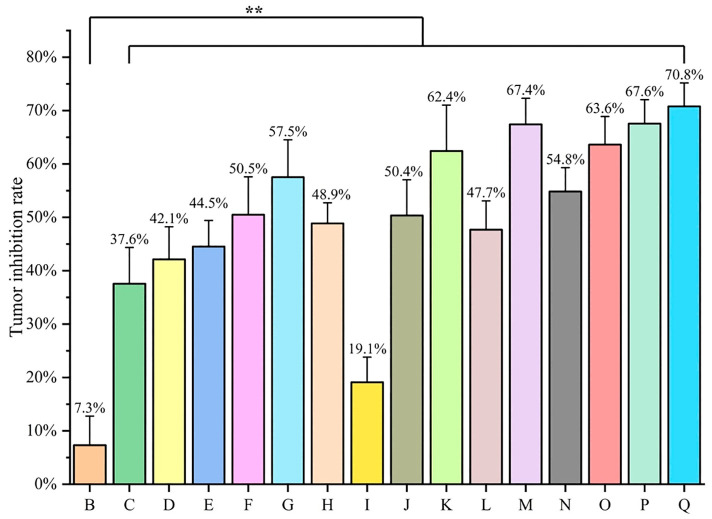
Summary of tumor inhibition rates of nude mice with pancreatic cancer tumors in different-gene siRNA-LNP administration groups. ** *p* < 0.01 (n = 7). From B to Q are in turn NC siRNA-LNP, TACSTD2 siRNA-LNP, SDC1 siRNA-LNP, CE 1.5 DDR1 (6:1) siRNA-LNP, DMG DDR1 (6:1) siRNA LNP 1 mg/kg, DMG DDR1 (6:1) siRNA LNP 2 mg/kg, CE 1.5 DDR1 (12:1) siRNA LNP, TGFβ-1 siRNA-LNP, TGFβ-1/DDR1 siRNA LNP, TACSTD2/DDR1 siRNA-LNP, SDC1/DDR1 siRNA-LNP, TACSTD2/TGFβ-1/DDR1 siRNA LNP, SDC1/TGFβ-1/DDR1 siRNA LNP, SDC1/TACSTD2/DDR1 siRNA LNP, SDC1/TACSTD2/TGFβ-1 siRNA LNP and SDC1/TACSTD2/TGFβ-1/DDR1 siRNA LNP.

Nude mice carrying peritoneal cancer in all groups were killed by spinal cord dislocation after drug administration, and their tumors were removed. The tumors of nude mice of all groups were photographed and weighed, and the tumor inhibition rate was calculated according to tumor weight data. The results of the tumor inhibition rate are illustrated in Figure 18. The tumor inhibition rate of the LNP administration group with different-gene siRNAs was significantly better than that of the NC siRNA-LNP group and the normal saline group.

#### 2.7.1. Comparison of Tumor Inhibition Rate in Nude Mice with a Pancreatic Cancer Tumor in a Single siRNA-LNP Administration Group

**Figure 19:**
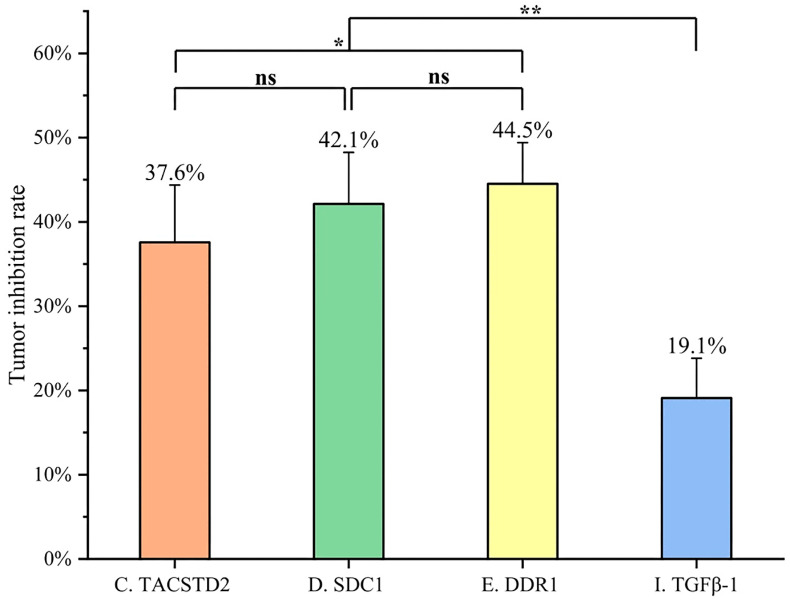
Comparison of tumor inhibition rates in nude mice with pancreatic cancer tumors in the single siRNA-LNP administration group. ** *p* < 0.01; * *p* < 0.05; ns means *p* > 0.05 (*n* = 7).

**Figure 20:**
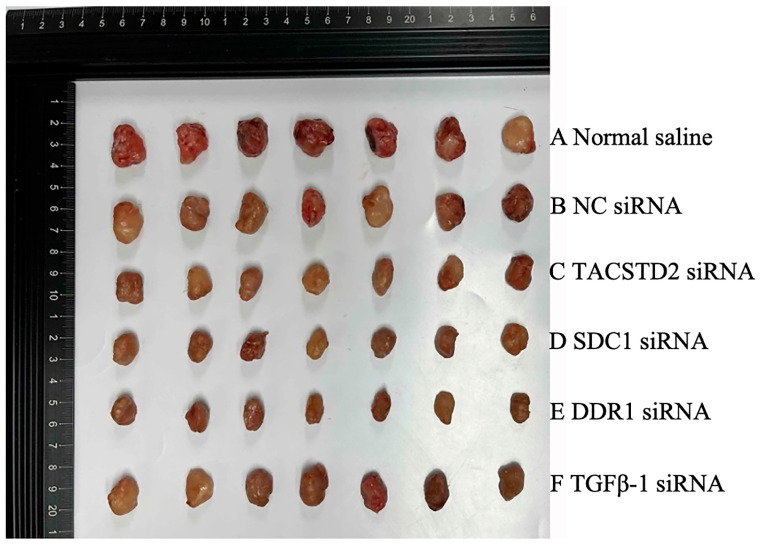
Tumor tissue of nude mice with pancreatic cancer in the LNP administration group with single siRNA (*n* = 7).

The tumor inhibition rate for the single siRNA-LNP administration group is illustrated in Figure 19, and the tumor tissue of tumor-bearing nude mice in the single siRNA-LNP administration group is depicted in Figure 20. The tumor inhibition rate of TACSTD2 siRNA-LNP, SDC1 siRNA-LNP, and DDR1 siRNA-LNP was significantly better than that of TGFβ-1 siRNA-LNP, and the difference was statistically significant. The tumor inhibition rates of TACSTD2 siRNA-LNP and SDC1 siRNA-LNP were similar, with statistically non-significant differences between them. SDC1 siRNA-LNP and DDR1 siRNA-LNP indicated significantly higher tumor inhibition rates than the comparison; the difference was insignificant. However, DDR1 siRNA-LNP was better than TACSTD2 siRNA-LNP, and the difference was statistically significant.

#### 2.7.2. Comparison of Tumor Inhibition Rates in Nude Mice with a Pancreatic Cancer Tumor in Different-Prescription DDR1 LNP Administration Groups

**Figure 21:**
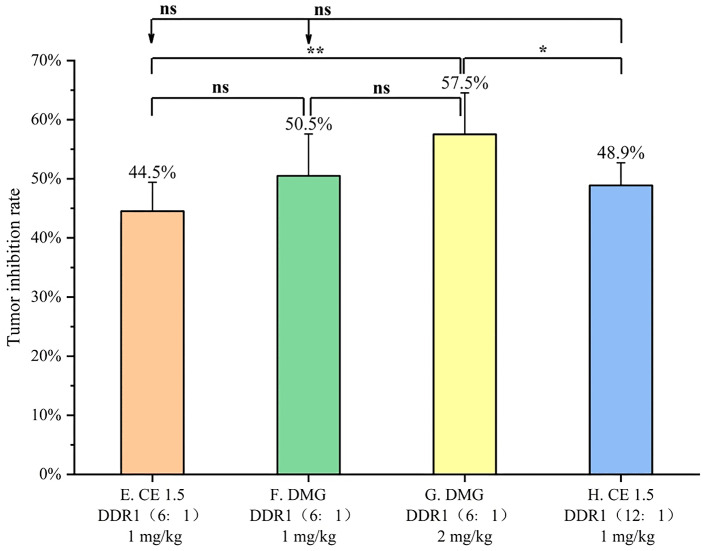
Comparison of tumor inhibition rates in nude mice with pancreatic cancer tumors in different DDR1 siRNA-LNP administration groups. ** *p* < 0.01; * *p* < 0.05; ns means *p* > 0.05 (*n* = 7).

**Figure 22:**
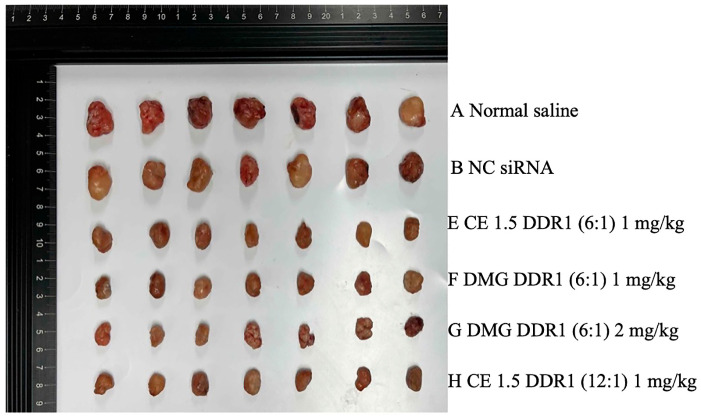
Tumor tissues of nude mice with pancreatic cancer in different-prescription DDR1 siRNA-LNP administration groups (*n* = 7).

The tumor inhibition rates of pancreatic tumor-bearing nude mice treated with DDR1 siRNA-LNP administration groups with different prescriptions are depicted in Figure 21, and the corresponding tumor tissues of nude mice bearing pancreatic cancer tumors in the DDR1 siRNA-LNP administration group with different prescriptions are illustrated in Figure 22. The tumor inhibition rate of CE 1.5 siRNA-LNP (6:1) 1 mg/kg was similar to that of CE 1.5 siRNA-LNP (12:1) 1 mg/kg and DMG siRNA-LNP (6:1) 1 mg/kg, with statistical non-significance. The tumor inhibition rate of DMG siRNA-LNP (6:1) 1 mg/kg was lower than that of DMG siRNA-LNP (6:1) 2 mg/kg, and the difference was statistically non-significant. The tumor inhibition rate of CE 1.5 siRNA-LNP (6:1) 1 mg/kg was lower than that of DMG siRNA-LNP 2 mg/kg, and the difference was statistically significant. The tumor inhibition rate of CE 1.5 siRNA-LNP (12:1) 1 mg/kg was lower than that of DMG siRNA-LNP 2 mg/kg, and the difference was statistically significant.

#### 2.7.3. Comparison of Tumor Inhibition Rates in Nude Mice of Pancreatic Cancer-Bearing Tumors in Single and Double siRNA-LNP Administration Groups

**Figure 23:**
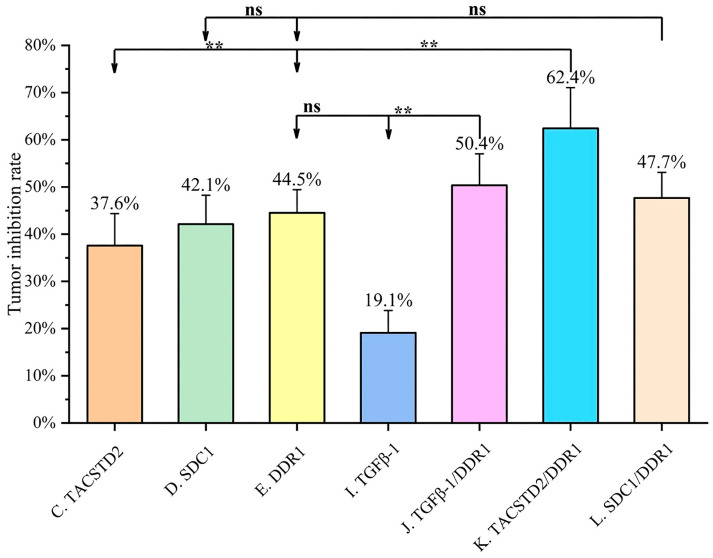
Comparison of tumor inhibition rates in nude mice with pancreatic cancer tumors in single and double siRNA-LNP administration groups. ** *p* <0.01; ns means *p* > 0.05 (*n* = 7).

The tumor inhibition rate of pancreatic tumor-bearing nude mice in the single and dual siRNA-LNP treatment groups is illustrated in Figure 23. The tumor inhibition rate of TGFβ-1/DDR1 siRNA-LNP was slightly stronger than that of DDR1 siRNA-LNP, and the difference was statistically non-significant. SDC1/DDR1 siRNA-LNP was slightly stronger than the DDR1 siRNA-LNP and SDC1 siRNA-LNP, and the difference was statistically non-significant. The tumor inhibition rate of TACSTD2/DDR1 siRNA-LNP was significantly better than that of TACSTD2 siRNA-LNP and DDR1 siRNA-LNP. The comparison difference was statistically significant, and the combined effect was obvious.

#### 2.7.4. Comparison of Tumor Inhibition Rate in Nude Mice with a Pancreatic Cancer Tumor in Multiple siRNAs Combined with LNP Administration Group

**Figure 24:**
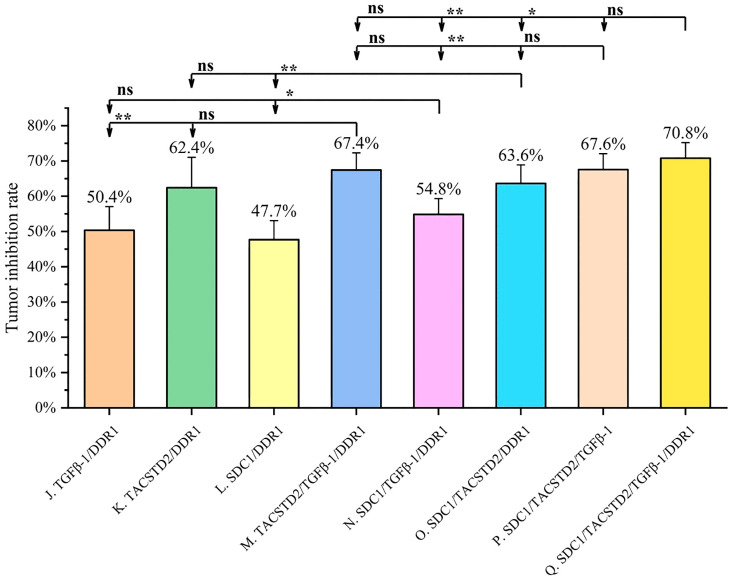
Comparison of tumor inhibition rates in nude mice with pancreatic cancer tumors in the multiple siRNAs combined with LNP administration group. ** *p* < 0.01; * *p* < 0.05; ns means *p* > 0.05 (*n* = 7).

**Figure 25:**
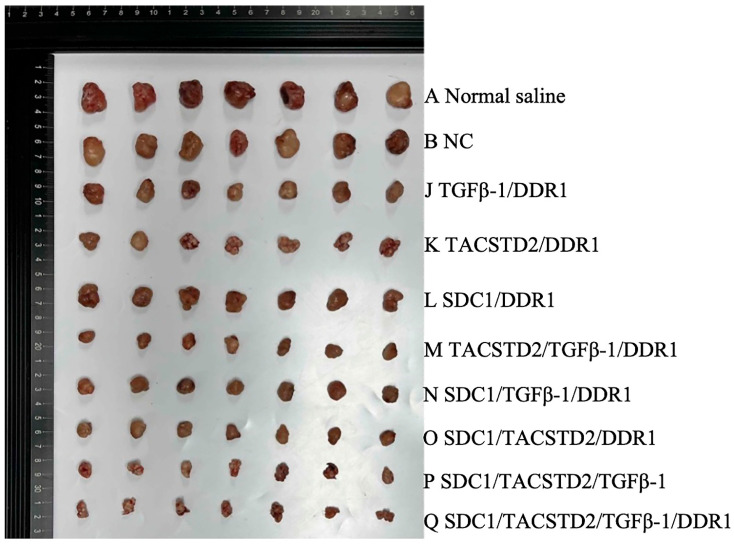
Tumor tissues of nude mice with pancreatic cancer in the multiple siRNAs combined with LNP administration group (*n* = 7).

The tumor inhibition rate of tumor-bearing nude mice in the multiple siRNA combined LNP administration group is illustrated in Figure 24, and the tumor tissue of tumor-bearing nude mice in the multiple siRNA combined LNP administration group is illustrated in Figure 25. The tumor inhibition rate of SDC1/TACSTD2/TGFβ-1 siRNA-LNP was superior to that of SDC1/TGFβ-1/DDR1 siRNA-LNP, and the difference was statistically significant. The tumor inhibition rate of TACSTD2/TGFβ-1/DDR1 siRNA-LNP was better than that of TGFβ-1/DDR1 siRNA-LNP, and the difference was statistically significant. The tumor inhibition rate of TACSTD2/TGFβ-1/DDR1 siRNA-LNP was slightly better than that of TACSTD2/DDR1 siRNA-LNP, and the comparison difference was statistically non-significant. SDC1/TACSTD2/TGFβ-1/DDR1 siRNA was superior or significantly superior to TGFβ-1/DDR1 siRNA-LNP, SDC1/DDR1 siRNA-LNP, TACSTD2/DDR1 siRNA-LNP, SDC1/TGFβ-1/DDR1 siRNA LNP, and SDC1/TACSTD2/DDR1 siRNA-LNP. The combined effect is obvious. The tumor inhibition rate of SDC1/TACSTD2/TGFβ-1/DDR1 siRNA-LNP was similar to that of TACSTD2/TGFβ-1/DDR1 siRNA-LNP and SDC1/TACSTD2/TGFβ-1 siRNA-LNP, with a statistically non-significant difference.

#### 2.7.5. Three or Four Kinds of siRNA Joint LNP Dosage Group Total Dose (1 mg/kg) and DMG DDR1 siRNA-LNP Inhibitory Rate Comparison of 2 mg/kg

**Figure 26:**
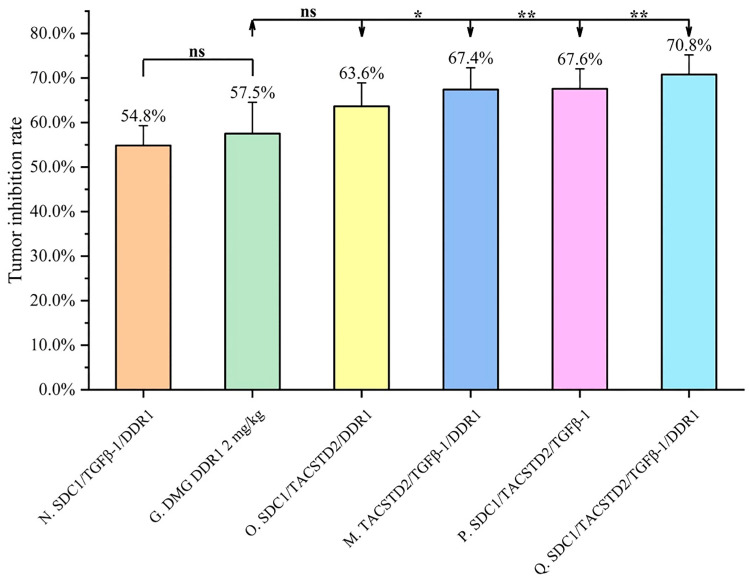
Comparison of tumor inhibition rates of the three and four kinds of siRNA combined with LNPs group (total dose: 1 mg/kg) and the DMG DDR1 siRNA-LNP group (2 mg/kg) in pancreatic cancer tumor-bearing nude mice. ** *p* < 0.01; * *p* < 0.05; ns means *p* > 0.05.

The tumor inhibition rates for groups treated with three- and four-siRNA-combined LNPs (1 mg/kg) and DMG DDR1 siRNA-LNP 2 mg/kg are illustrated in Figure 26. The tumor inhibition rates of three- and four-siRNA-combined LNPs (total dose of all 1 mg/kg) were compared with that of DMG DDR1 siRNA-LNP 2 mg/kg, There was a statistically non-significant difference between the inhibitory rates of the DMG DDR1 siRNA-LNP 2 mg/kg and SDC1/TACSTD2/DDR1 siRNA-LNP. The tumor inhibition rate of DMG DDR1 siRNA-LNP 2 mg/kg was similar to that of SDC1/TGFβ-1/DDR1 siRNA-LNP, with a statistically non-significant difference. The tumor inhibition rate of TACSTD2/TGFβ-1/DDR1 siRNA-LNP was better than that of DMG DDR1 siRNA-LNP 2 mg/kg, and the difference was statistically significant. The tumor inhibition rates of SDC1/TACSTD2/TGFβ-1 siRNA-LNP and SDC1/TACSTD2/TGFβ-1/DDR1 siRNA-LNP were significantly better than DMG DDR1 siRNA-LNP 2 mg/kg, and the difference was statistically significant. The results indicated that the tumor inhibition rate of three siRNAs combined with LNPs and four siRNAs combined with LNPs (total dose of 1 mg/kg) was similar to or higher than that when doubling the dose of a single siRNA-LNP (2 mg/kg). The combined effect was apparent, indicating that multiple siRNAs combined with LNPs could significantly improve efficacy, surpassing that of a high-dose single siRNA-LNP.

### 2.8. Pathological Section Analysis

The nude mice with pancreatic cancer were killed immediately after administration, and the tumor, heart, liver, spleen, lung, kidney, and stomach organs were surgically dissected for pathological section analysis.

**Figure 27:**
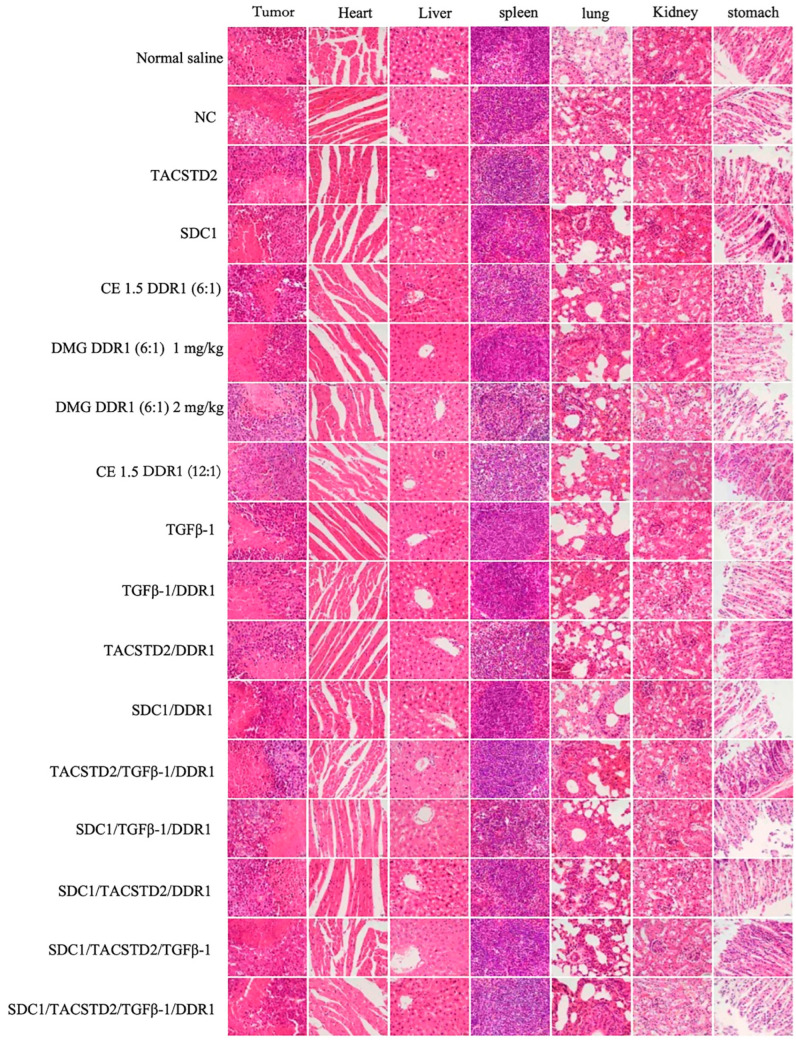
Pathological sections of tumors and organs in nude mice with pancreatic cancer (*n* = 7).

The pathological section analysis results of pancreatic cancer tumor-bearing nude mice in different-gene siRNA-LNP administration groups were scored according to the scoring criteria, as depicted in Figure 27. The results indicated that the tumor pathological section analysis results were similar for all gene siRNA-LNP administration groups, but the necrotic area was slightly different. The cardiac injury in the LNP administration group of all gene siRNA was slightly higher than that in the normal saline group, which was similar to that in the NC siRNA-LNP group, indicating that the excipient, mainly DLin-MC3-DMA, may aggravate the cardiac injury. Excipients in LNPs had slight effects on the heart and liver. In the LNP administration group, the structure of hepatic lobules was disturbed, and the inflammation around hepatic lobule vessels and in hepatic parenchyma was slightly aggravated than that in the normal saline group, indicating that LNPs with different genes of siRNA had specific effects on the liver. The spleen changes in the siRNA-LNP administration group were the same as in the normal saline group. Alveolar wall thickening and lung tissue congestion were observed in all gene siRNA-LNP administration groups. Excipients and drugs had slight effects on the lungs. Renal tubule swelling, renal interstitial edema, and congestion were slightly heavier in the siRNA-LNP administration group compared to the normal saline group. Drugs had a slight effect on the kidney, while excipients had no obvious effect on the kidney. In the LNP administration group of most genes siRNA, gastric mucosal epithelial exfoliation was intensified compared with the normal saline group. There was inflammatory cell infiltration in the mucosal layer, and drugs slightly affected the stomach. Excipients and different-gene siRNA-LNP administration groups did not affect the spleen. Generally, the toxicity of various siRNA-LNP genes to the organs of nude mice with pancreatic cancer was little and the LNP genes had specific effects on the liver, lung, kidney, and stomach. LNP excipients may primarily be DLin-MC3-DMA, particularly affecting the heart, liver, and lungs.

## 3. Results

This study constructed a siRNA-LNP formulation system using PEG CE as a stabilizer. The DDR1/TGFβ-1/TACSTD2/SDC1 siRNA co-loaded LNP had a small particle size and high gene inhibition efficiency. Multiple siRNAs loaded into the same LNP can inhibit the expression of multiple tumor-related genes simultaneously, play a combined effect, and significantly enhance the efficacy of treating pancreatic cancer. The combination of multiple siRNAs with LNPs was slightly toxic, and the pros and cons of efficacy and toxicity should be measured. However, as pancreatic cancer is challenging to treat and has a high fatality rate, slight drug toxicity is usually acceptable.

Other parts of the experiment are shown in the Supplementary Materials.

## 4. Discussion

We evaluated the antitumor efficacy of LNPs with different siRNA genes in nude mice with pancreatic cancer. The results indicated that DDR1 siRNA-LNP, TGFβ-1 siRNA-LNP, TACSTD2 siRNA-LNP, and SDC1 siRNA-LNP have significant efficacy in the treatment of pancreatic cancer tumor-bearing nude mice, and DDR1 siRNA-LNP has the best efficacy among single siRNA-LNP. The combined efficacy of three and four kinds of siRNA-LNP was stronger than that of single siRNA-LNP, and the combined effect was noticeable. The tumor inhibition rate of the LNPs combined with three siRNAs is slightly lower than that of the combined LNP of four siRNAs. Its safety is generally acceptable, but attention should be paid to the toxicity caused by LNP excipients, especially cationic lipids. It also suggests that the combination of multiple siRNAs may improve the efficacy of other tumors, which also has good reference significance for achieving the combination of multiple siRNAs for the treatment of multiple tumors.

## Figures and Tables

**Table 1:** The particle size of siRNA-LNPs.

Prescription	Different-Gene siRNA-LNPs	Mean Size (nm)	SD (nm)	PDI
DMG	NC	100.7 ± 4.5	36.3 ± 2.0	0.130 ± 0.003
DMG	DDR1	97.6 ± 4.1	22.6 ± 5.6	0.055 ± 0.023
CE 1.5	NC	82.0 ± 3.1	28.6 ± 3.2	0.122 ± 0.024
CE 1.5	TACSTD2	81.9 ± 3.2	31.3 ± 3.9	0.146 ± 0.026
CE 1.5	SDC1	84.5 ± 1.7	26.9 ± 1.7	0.102 ± 0.011
CE 1.5	TGFβ-1	83.1 ± 1.3	23.5 ± 7.5	0.085 ± 0.049
CE 1.5	DDR1	82.9 ± 1.0	25.7 ± 1.8	0.097 ± 0.012
CE 1.5	DDR1 (12:1)	78.1 ± 2.3	34.5 ± 4.7	0.197 ± 0.041
CE 1.5	TGFβ-1/DDR1	82.0 ± 1.5	27.6 ± 5.3	0.115 ± 0.042
CE 1.5	TACSTD2/DDR1	82.3 ± 1.0	24.7 ± 3.7	0.092 ± 0.029
CE 1.5	SDC1/DDR1	84.8 ± 0.7	27.6 ± 2.1	0.106 ± 0.017
CE 1.5	TACSTD2/TGFβ-1/DDR1	84.1 ± 1.9	28.3 ± 9.0	0.119 ± 0.064
CE 1.5	SDC1/TGFβ-1/DDR1	81.2 ± 1.0	25.4 ± 2.8	0.099 ± 0.02
CE 1.5	SDC1/TACSTD2/DDR1	82.8 ± 1.7	29.2 ± 2.6	0.125 ± 0.017
CE 1.5	SDC1/TACSTD2/TGFβ-1	82.8 ± 0.9	28.9 ± 2.0	0.122 ± 0.014
CE 1.5	SDC1/TACSTD2/TGFβ-1/DDR1	82.2 ± 0.4	27.1 ± 6.5	0.112 ± 0.049

**Table 2:** The charge of siRNA-LNPs.

Prescription	Different-Gene siRNA-LNPs	pH 4 Medium Potential (mV)	pH 7.4 Medium Potential (mV)
DMG	NC	11.46 ± 1.12	−13.86 ± 0.93
DMG	DDR1	14.69 ± 1.03	−11.06 ± 0.92
CE 1.5	NC	3.62 ± 0.74	−14.91 ± 1.08
CE 1.5	TACSTD2	4.76 ± 1.13	−14.42 ± 0.54
CE 1.5	SDC1	5.55 ± 0.35	−11.80 ± 1.09
CE 1.5	TGFβ-1	4.73 ± 0.69	−12.35 ± 0.58
CE 1.5	DDR1	4.51 ± 0.80	−14.23 ± 0.54
CE 1.5	DDR1 (12:1)	7.37 ± 0.18	−13.27 ± 0.24
CE 1.5	TGFβ-1/DDR1	4.66 ± 0.17	−12.15 ± 1.09
CE 1.5	TACSTD2/DDR1	4.56 ± 0.40	−13.46 ± 0.99
CE 1.5	SDC1/DDR1	4.47 ± 1.40	−12.21 ± 0.27
CE 1.5	TACSTD2/TGFβ-1/DDR1	4.64 ± 0.81	−13.47 ± 1.59
CE 1.5	SDC1/TGFβ-1/DDR1	4.09 ± 0.47	−12.26 ± 0.69
CE 1.5	SDC1/TACSTD2/DDR1	4.31 ± 0.29	−11.76 ± 0.89
CE 1.5	SDC1/TACSTD2/TGFβ-1	4.50 ± 0.41	−8.40 ± 1.25
CE 1.5	SDC1/TACSTD2/TGFβ-1/DDR1	4.41 ± 0.30	−5.02 ± 2.04

## Data Availability

Data are contained within the article and Supplementary Materials.

## Supplementary Materials

The following supporting information can be downloaded at: https://www.mdpi.com/article/10.3390/pharmaceutics18070775/s1. Figure S1. mRNA expression level of PaTu 8988 cells treated with PBS (lipid–nucleic acid ratio 6:1); Figure S2. mRNA expression levels of pancreatic cancer PaTu 8988 cells treated with different-prescriptions of DDR1 siRNA-LNP (lipid–nucleic acid ratio 6:1); Figure S3. Comparison of mRNA expression inhibition rates of pancreatic cancer PaTu 8988 cells treated with different-prescription DDR1 siRNA-LNPs (lipid–nucleic acid ratio 6:1); Figure S4. mRNA expression levels of pancreatic cancer PaTu 8988 cells treated with different TGFβ-1 siRNA-LNP prescriptions (lipid–nucleic acid ratio 6:1); Figure S5. Comparison of mRNA expression inhibition rates of pancreatic cancer PaTu 8988 cells treated with different TGFβ-1 siRNA-LNP prescriptions (lipid–nucleic acid ratio 6:1); Figure S6. mRNA expression level of pancreatic cancer PaTu 8988 cells treated with different TACSTD2 siRNA-LNP prescriptions (lipid–nucleic acid ratio 6:1); Figure S7. Comparison of mRNA expression inhibition rates of pancreatic cancer PaTu 8988 cells treated with different TACSTD2 siRNA-LNP prescriptions (lipid–nucleic acid ratio 6:1); Figure S8. mRNA expression levels of pancreatic cancer PaTu 8988 cells treated with different SDC1 siRNA-LNP prescriptions (lipid–nucleic acid ratio 6:1); Figure S9. Comparison of mRNA expression inhibition rates of pancreatic cancer PaTu 8988 cells treated with different SDC1 siRNA-LNP prescriptions (lipid–nucleic acid ratio 6:1); Figure S10. Average fluorescence intensity of 2 h uptake of DDR1 siRNA-LNP with different prescriptions and different lipid–nucleic acid ratios; Figure S11. Average fluorescence intensity of LNP of DDR1 siRNA with different prescriptions and different lipid–nucleic acid ratios at 6 h; Figure S12. The average fluorescence intensity of DDR1 siRNA-LNP ingested at 18 h with different prescriptions and different lipid–nucleic acid ratios; Figure S13. Summary of RTV of cancer-bearing nude mice of different-gene siRNA-LNP administration groups; Figure S14. Comparison of RTV in nude mice with pancreatic cancer in the single siRNA-LNP administration group; Figure S15. Comparison of RTV in nude mice with pancreatic cancer tumors in different DDR1 siRNA-LNP administration groups; Figure S16. Comparison of RTV in nude mice with pancreatic cancer in the single and double siRNA-LNP administration groups; Figure S17. Comparison of RTV in nude mice with pancreatic cancer tumors in multiple siRNAs combined with LNP administration group; Figure S18. Comparison of RTV in nude mice with pancreatic cancer tumors in the three and four siRNA combined LNP administration group (total dose of 1 mg/kg) and DMG DDR1 siRNA-LNP administration group (2 mg/kg); Figure S19. T/C summary of pancreatic cancer tumor-bearing nude mice in different-gene siRNA-LNP administration groups; Figure S20. Effects of LNP administration groups with different siRNA genes on body weight of nude mice bearing pancreatic cancer.

## Abbreviations

The following abbreviations are used in this manuscript:

siRNA-LNP	siRNA lipid nanoparticles
DDR1	Disk domain receptor 1
TGFβ1	Transforming growth factor β1
TACSTD2	Tumor-associated calcium signal transduction protein 2
SDC1	Polyligand proteoglycan 1
