# Supplementary material for: Study on Synergistic Treatment of Pancreatic Cancer by Multiple Small Interfering Ribonucleic Acid Lipid Nanoparticles of Disk Domain Receptor 1, Transforming Growth Factor β1, Tumor-Associated Calcium Signal Transduction Protein 2, and Polyligand Proteoglycan 1
Source: Pharmaceutics. 2026 Jun 25;18(7):775. doi: 10.3390/pharmaceutics18070775 (PMC13414619; doi:10.3390/pharmaceutics18070775)
Supplement: Supplementary file 1 [file pharmaceutics-18-00775-s001.zip › pharmaceutics-4356595-supplementary.pdf]

Supplementary Material

Methods for Detecting mRNA Gene Expression Level

To study the inhibitory effect of DDR1 siRNA LNP, TGFβ-1 siRNA LNP, TACSTD2 siRNA LNP, and SDC1 siRNA LNP on mRNA-related genes of pancreatic cancer PaTu 8988 cells, quantitative PCR (qPCR) method was used to detect the mRNA expression levels of pancreatic cancer PaTu 8988 cell-related genes treated with different gene siRNA LNP of each prescription. This allowed the evaluation of the inhibitory effect of varying gene siRNA LNPs for each prescription on pancreatic cancer PaTu 8988 cell-related genes' mRNA.

qPCR

Pancreatic cancer PaTu 8988 cells were inoculated in a 6-well plate culture dish at a density of 5×10^5/dish and incubated overnight with 5 mL medium. When the cells reached approximately 60% at the bottom of the dish, they were divided into groups with medicine and cultured for 24 and 48 h. The RNA was extracted using the instructions of the FastPure® Cell/Tissue Total RNA Isolation Kit V2, and the extracted RNA was directly used for reverse transcription into complementary deoxyribonucleic acid (cDNA). The reaction system was prepared according to the instructions of the HiScript® III All-in-one RT SuperMix Perfect for qPCR kit for reverse transcription reaction. The reverse transcription reaction conditions were 50°C, 15 min; 85°C, 5 s; real-time fluorescence qPCR reaction was performed on the cDNA generated by reverse transcription. The reaction system was prepared with the instructions of the SYBR Green Pro Taq HS premixed qPCR kit, and the prepared reaction system was added to the 96-well plate for real-time fluorescence qPCR reaction. The reaction conditions of real-time fluorescence qPCR were as follows: 95°C, 30 s pre-denatured; 95°C, 5 s; 60°C, 30 s, 40 cycles of reaction; 95°C, 5 s; 65°C, 60 s; 95°C, 1 s melting curve. Statistical software GraphPad Prism (version 9.0.0) was used for data analysis.

Western Blot

Pancreatic cancer PaTu 8988 cells were inoculated in a 6-well plate culture dish at a density of 5×10^5/dish and incubated overnight with 5 mL medium. When the cells reached approximately 60% at the bottom of the dish, they were divided into groups with medicine and cultured for 72 h to extract protein. According to several samples, an appropriate amount of BCA working liquid was prepared at a ratio of 50:1 according to the instructions and thoroughly mixed for protein quantification. A total of 12% separation glue and 5% concentrated glue were prepared according to the formula. The concentrated glue was injected into the upper end of the separation glue, and the corresponding sample comb was inserted. Following glue solidification, the comb was pulled out, and 1× Tris-Gly electrophoresis buffer was added. A volume of 25 μL of sample supernatant was absorbed and added into the sample hole, with 5 μL of protein marker added into the hole beside the sample. The power supply was turned on, and the voltage was set to 60 V initially. When the protein sample entered the separation gel, the voltage could be increased to 90 V. Regarding the position of the pre-dyed marker, when the target strip entered the optimal separation zone of the gel, electrophoresis was stopped, and the film was transferred. After the transfer, the film was washed thrice with phosphate-buffered saline with Tween 20 (PBST) for 10 min each time. The film was put into the incubator box containing a monoclonal antibody diluent and incubated overnight with shaking at 4 °C, then removed on the second day and washed with tris-buffered saline with Tween 20 (TBST) 3 times for 10 min each time. Subsequently, the secondary antibody was diluted with 5% skimmed milk powder sealing solution, shaken at room temperature for 1 h, and washed with TBST 3 times for 5 min each time. Two kinds of enhanced chemiluminescence luminescent reagents, A and B, were mixed in equal volumes of 1:1, and the polyvinylidene difluoride membranes were covered with an AB mixture and fully contacted. Subsequently, the instrument

and computer were turned on, and Chemi capture software was opened. Automatic exposure and point shooting were selected to save the images. Data analysis was conducted using GraphPad Prism software (version 9.0.0).

mRNA Gene Expression Level Detection Results

PBS was added to culture cells as a baseline control and compared with NC siRNA LNP, as depicted in Figure S1. NC siRNA LNP did not inhibit mRNA expression in pancreatic cancer PaTu 8988 cells, and the difference between the NC siRNA LNP group and the PBS group was highly statistically significant. This indicates that NC siRNA LNP (which includes negative control siRNA and excipients) did not affect gene expression. Therefore, it can be used as a negative control for the prescription of different gene siRNA LNPs.

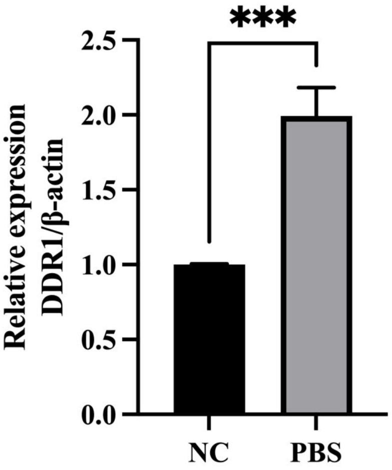

Figure S1. mRNA expression level of PaTu 8988 cells treated with NC siRNA LNP and PBS (lipid nucleic acid ratio 6:1). *** $P < 0.001$ ($n = 3$)

The mRNA expression levels of pancreatic cancer PaTu 8988 cells treated with different prescriptions of DDR1 siRNA LNP (6:1) are indicated in Figure S2. The mRNA expression inhibition rates of pancreatic cancer PaTu 8988 cells treated by DDR1 siRNA LNP with different dosage concentrations and culture times were compared, as depicted in Figure S3. Compared to the NC siRNA LNP, the mRNA expression of pancreatic cancer PaTu 8988 cells treated with different prescriptions of DDR1 siRNA LNP was significantly decreased, and the comparison differences were highly statistically significant. DDR1 siRNA LNP with different prescriptions could effectively inhibit the mRNA expression of pancreatic cancer PaTu 8988 cells. In the prescription of CE, although the dosage of CE is relatively high, it may gradually shed from the LNP over time to remove the influence on siRNA transfection. Considering that LNP undergoes blood circulation, CE may gradually shed during this period. Therefore, an appropriate increase in CE dosage may not affect the efficacy of siRNA LNP *in vivo*. Compared with NC siRNA LNP, mRNA expression of pancreatic cancer PaTu 8988 cells treated with different prescriptions of TGF β-1 siRNA LNP was significantly decreased, and the comparison differences were highly statistically significant. The mRNA expression level and inhibition rate are indicated in Figures S4-S5, respectively. Compared with the LNP of NC siRNA, the mRNA expression of pancreatic cancer PaTu 8988 cells treated with different prescriptions of TACSTD2 siRNA LNP was significantly decreased, and the comparison differences were highly statistically significant, as indicated in Figure S6 for mRNA expression level and Figure S7 for mRNA expression inhibition rate.

Compared with the LNP of NC siRNA, the mRNA expression of pancreatic cancer PaTu 8988 cells treated with different prescriptions of SDC1 siRNA LNP was significantly decreased, and the comparison differences were highly statistically significant, as depicted in Figure S8 for mRNA expression level and Figure S9 for mRNA expression inhibition rate. SDC1 siRNA LNP of the three prescriptions could effectively inhibit mRNA expression, and the inhibition effect was similar, but the difference in inhibition rate was statistically non-significant.

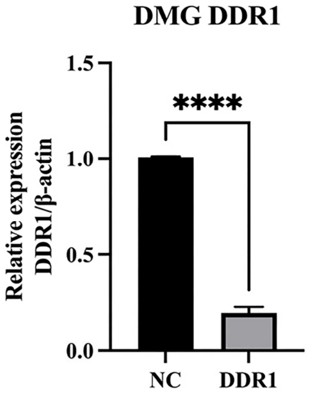

DMG DDR1, 2 μg/mL for 24 h

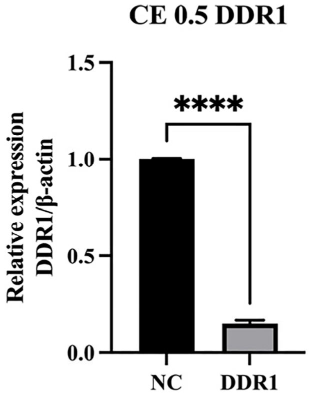

CE 0.5 DDR1, 2 μg/mL for 24 h

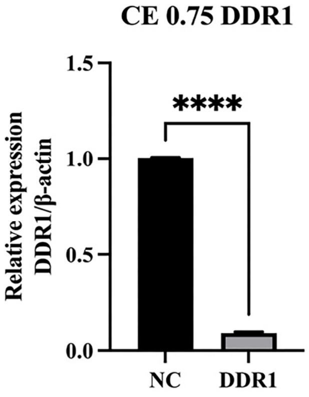

CE 0.75 DDR1, 4 μg/mL for 48 h

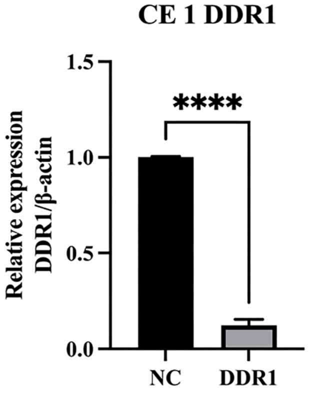

CE 1 DDR1, 2 μg/mL for 24 h

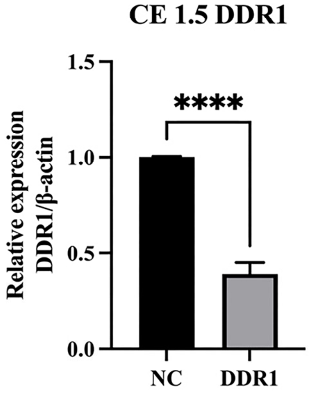

CE 1.5 DDR1, 2 μg/mL for 24 h

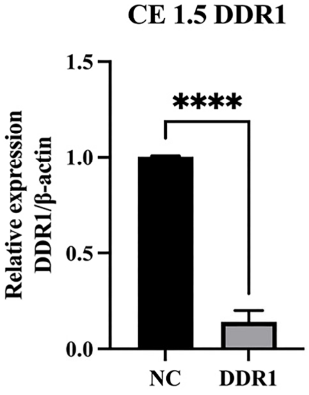

CE 1.5 DDR1, 4 μg/mL for 48 h

Figure S2. mRNA expression levels of pancreatic cancer PaTu 8988 cells treated with different prescriptions of DDR1 siRNA LNP (lipid nucleic acid ratio 6:1). **** $P < 0.0001$ ($n = 3$)

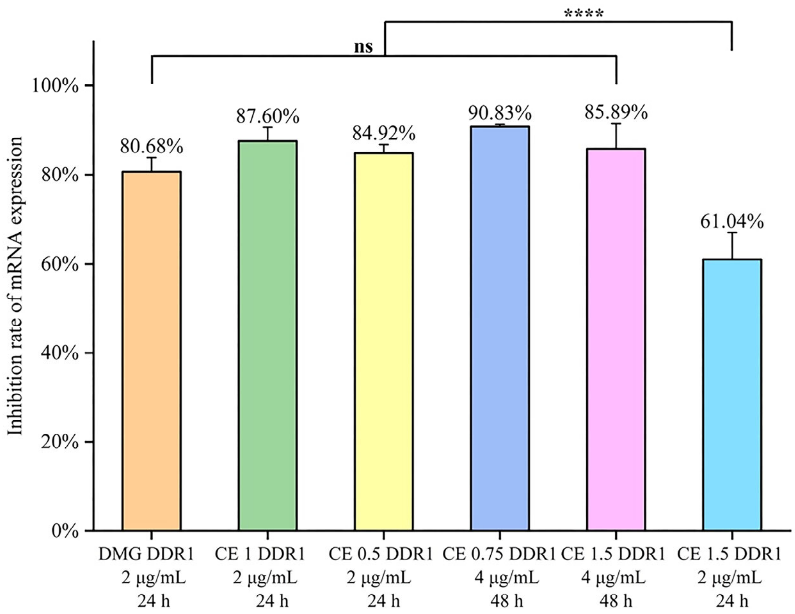

Figure S3. Comparison of mRNA expression inhibition rates of pancreatic cancer PaTu 8988 cells treated with different prescription DDR1 siRNA LNPs (lipid nucleic acid ratio 6:1). *** $P < 0.001$; ns means $P > 0.05$ ($n = 3$)

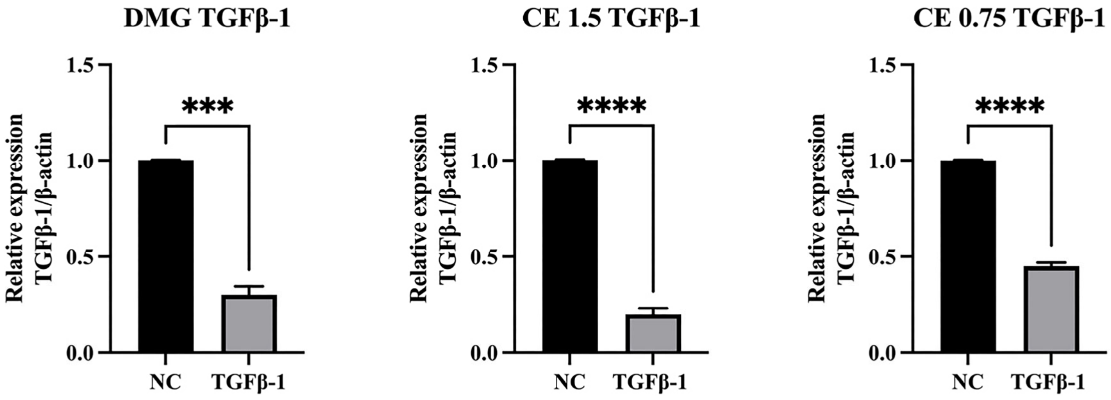

DMG TGFβ-1, 2 μg/mL for 24 h   CE 1.5 TGFβ-1, 4 μg/mL for 48 h   CE 0.75 TGFβ-1, 4 μg/mL for 48 h

Figure S4. mRNA expression levels of pancreatic cancer PaTu 8988 cells treated with different TGFβ-1 siRNA LNP prescriptions (lipid nucleic acid ratio 6:1). **** $P < 0.0001$; *** $P < 0.001$ ($n = 3$)

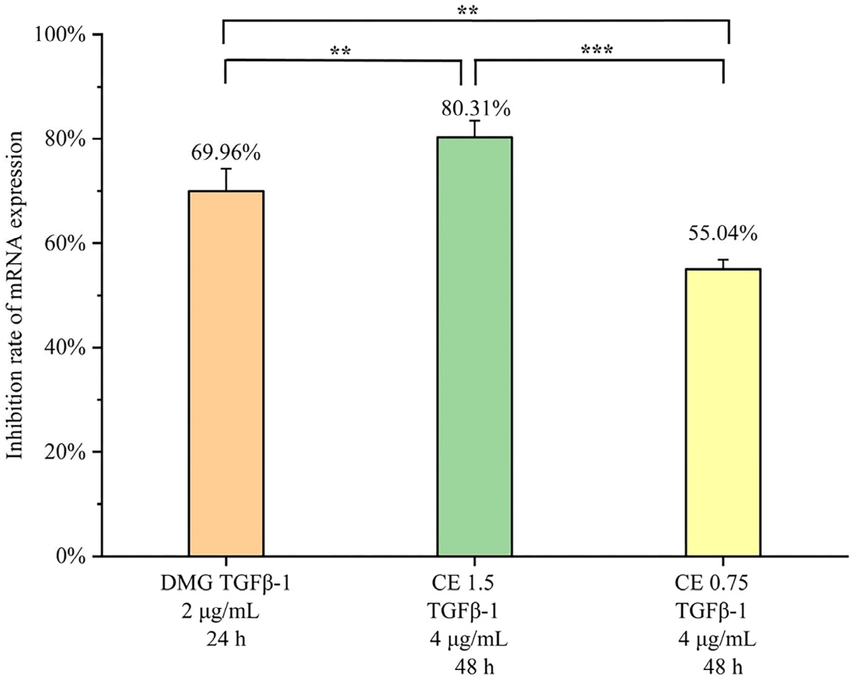

Figure S5. Comparison of mRNA expression inhibition rates of pancreatic cancer PaTu 8988 cells treated with different TGFβ-1 siRNA LNP prescriptions (lipid nucleic acid ratio 6:1). *** $P < 0.001$; ** $P < 0.01$; * $P < 0.05$ ($n = 3$)

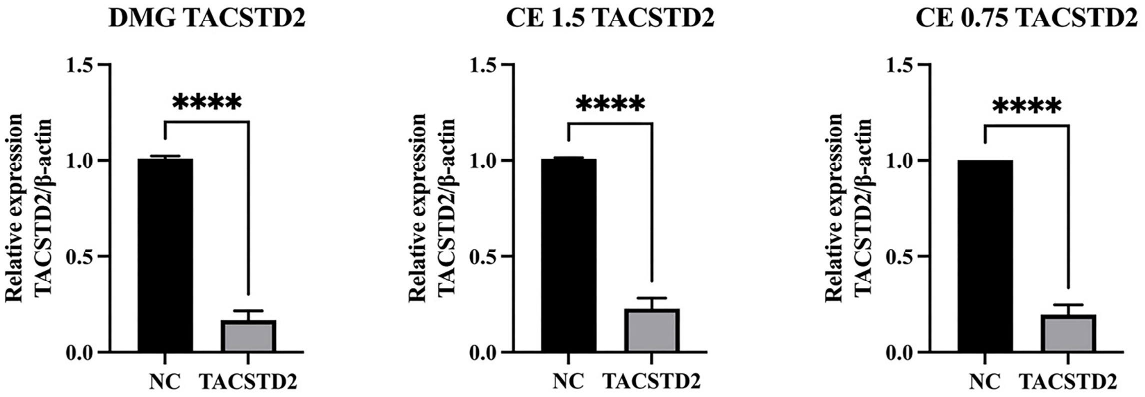

DMG TACSTD2, 3 μg/mL for 48 h

CE 1.5 TACSTD2, 3 μg/mL 48 h

CE 0.75 TACSTD2, 3 μg/mL 48 h

Figure S6. mRNA expression level of pancreatic cancer PaTu 8988 cells treated with different TACSTD2 siRNA LNP prescriptions (lipid nucleic acid ratio 6:1). **** $P < 0.0001$; *** $P < 0.001$ ($n = 3$)

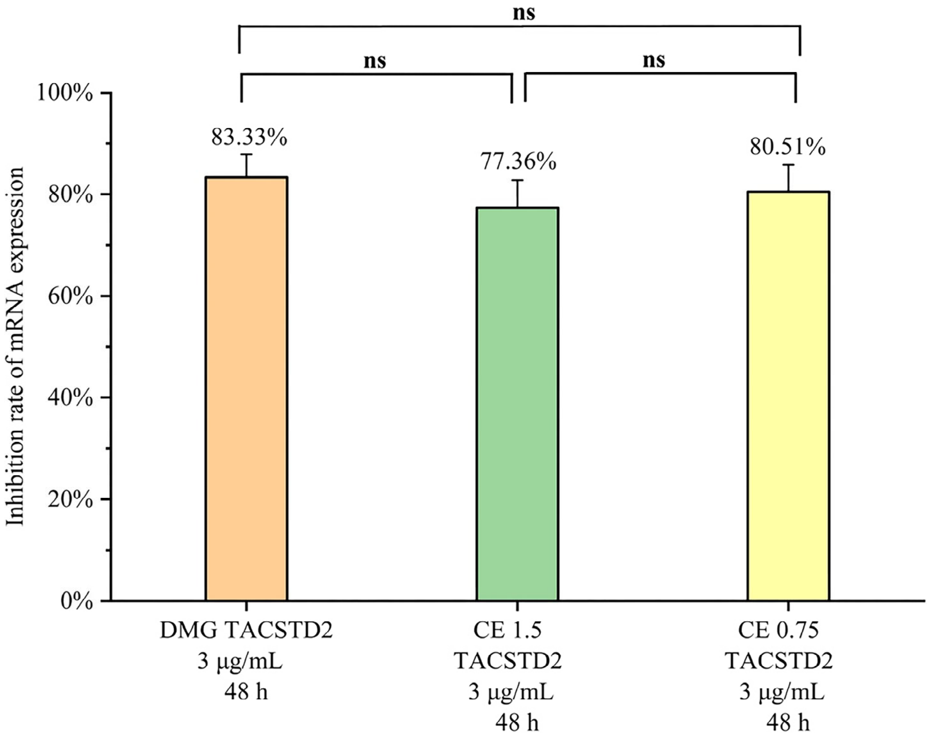

Figure S7. Comparison of mRNA expression inhibition rates of pancreatic cancer PaTu 8988 cells treated with different TACSTD2 siRNA LNP prescriptions (lipid nucleic acid ratio 6:1). ns means $P > 0.05$ ($n = 3$)

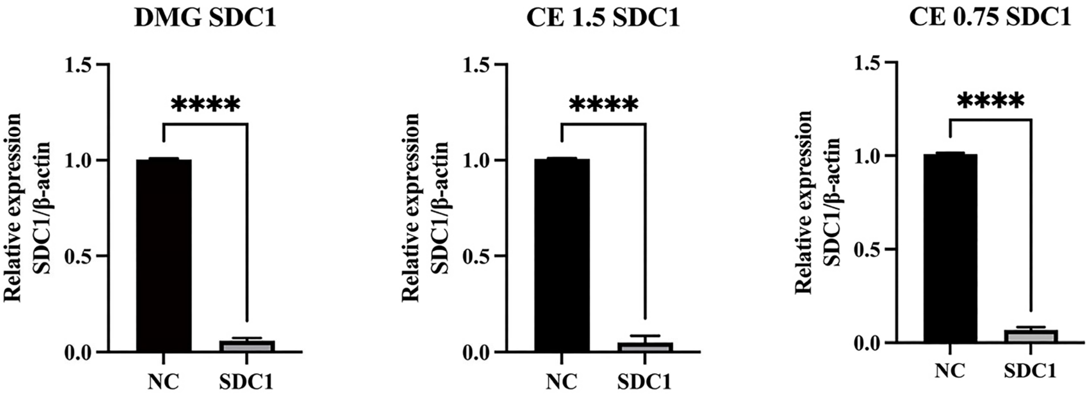

DMG SDC1, 2 μg/mL for 24 h   CE 1.5 SDC1, 4 μg/mL for 48 h   CE 0.75 SDC1, 4 μg/mL for 48 h

Figure S8. mRNA expression levels of pancreatic cancer PaTu 8988 cells treated with different SDC1 siRNA LNP prescriptions (lipid nucleic acid ratio 6:1). **** $P < 0.0001$ ($n = 3$)

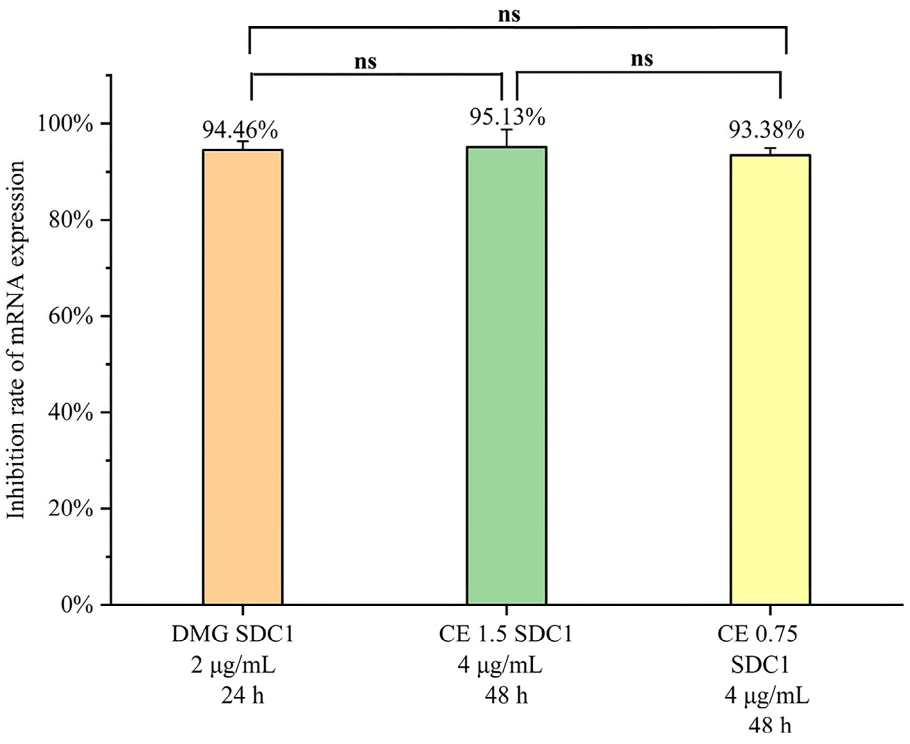

Figure S9. Comparison of mRNA expression inhibition rates of pancreatic cancer PaTu 8988 cells treated with different SDC1 siRNA LNP prescriptions (lipid nucleic acid ratio 6:1). ns means $P > 0.05$ ($n = 3$)

The average fluorescence intensity results of different prescriptions of 5' FAM DDR1 siRNA LNP ingested in PaTu 8988 cells at 2, 6, and 18 h were compared, as depicted in Figures S10–S12. At 2 and 6 h, the uptake of DMG DDR1 siRNA LNP (6:1), CE 1.5 DDR1 siRNA LNP (6:1), and CE 0.75 DDR1 siRNA LNP (6:1) was significantly different, but with the prolongation of time, the uptake of cells tended to be similar. At 2, 6, and 18 h, the uptake of DMG DDR1 siRNA LNP (12:1) was significantly different, while the uptake of CE 1.5 DDR1 siRNA LNP (12:1) and CE 0.75 DDR1 siRNA LNP (12:1) was less different. There were significant uptake differences among different lipid nucleic acids compared with DMG DDR1 siRNA LNP at 2 and 6 h, but non-significant uptake differences at 18 h, indicating that the uptake differences of DMG DDR1 siRNA LNP at different lipid nucleic acid ratios were small. There were significant uptake differences among different lipid nucleic acids compared with CE 1.5 DDR1 siRNA LNP at 2, 6, and 18 h, indicating that the uptake of varying lipid nucleic acids compared with CE 1.5 DDR1 siRNA LNP was significantly different.

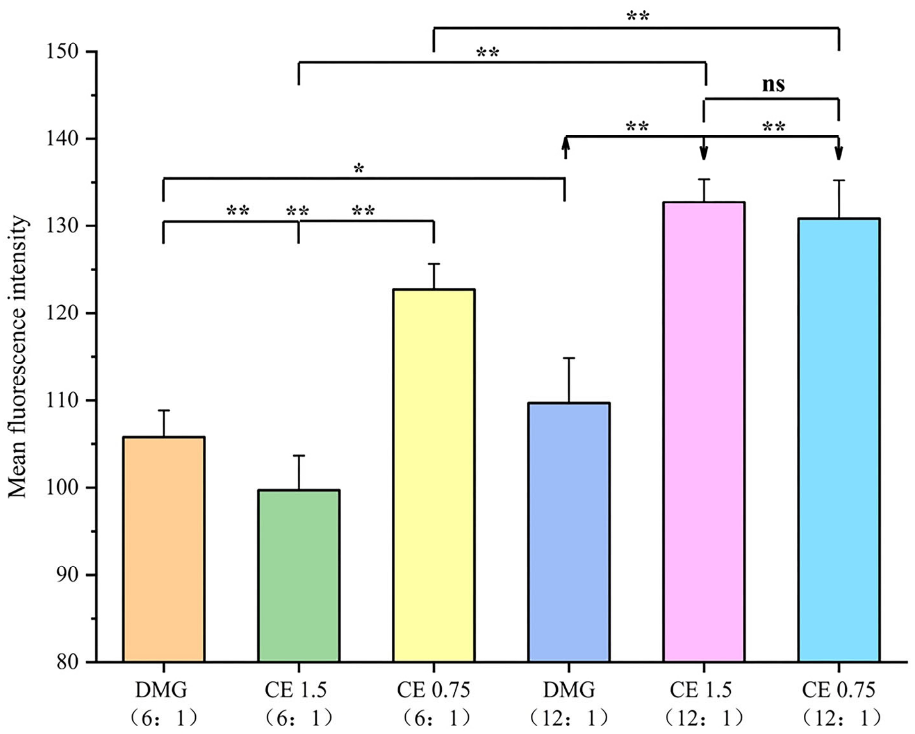

Figure S10. Average fluorescence intensity of 2 h uptake of DDR1 siRNA LNP with different prescriptions and different lipid nucleic acid ratios. ** $P < 0.01$; * $P < 0.05$; ns means $P > 0.05$ ($n = 3$)

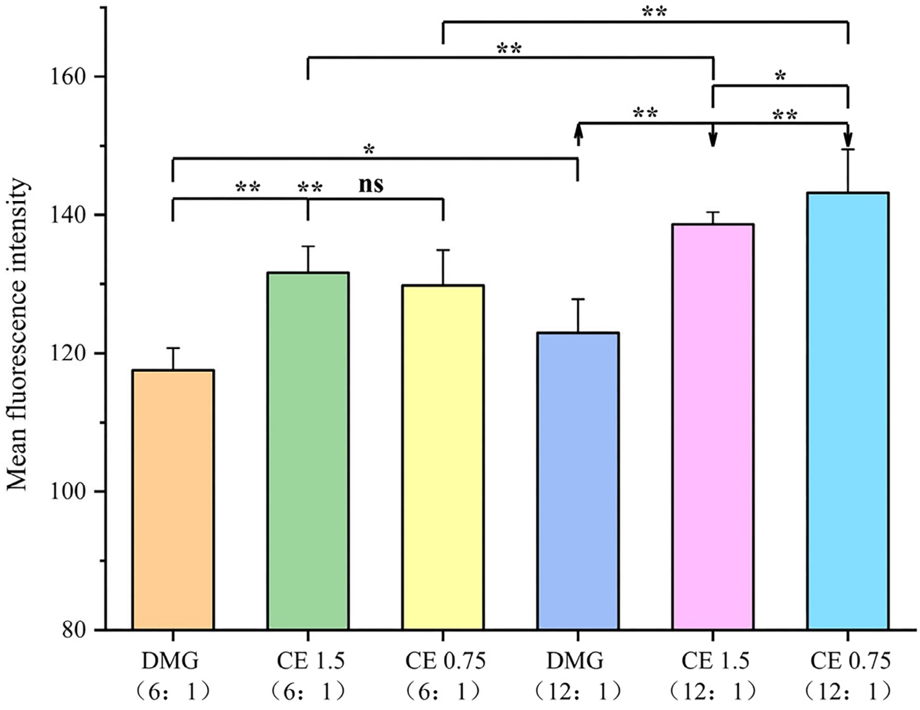

Figure S11. Average fluorescence intensity of LNP of DDR1 siRNA with different prescriptions and different lipid nucleic acid ratios at 6 h. ** $P < 0.01$; * $P < 0.05$; ns means $P > 0.05$ ($n = 3$)

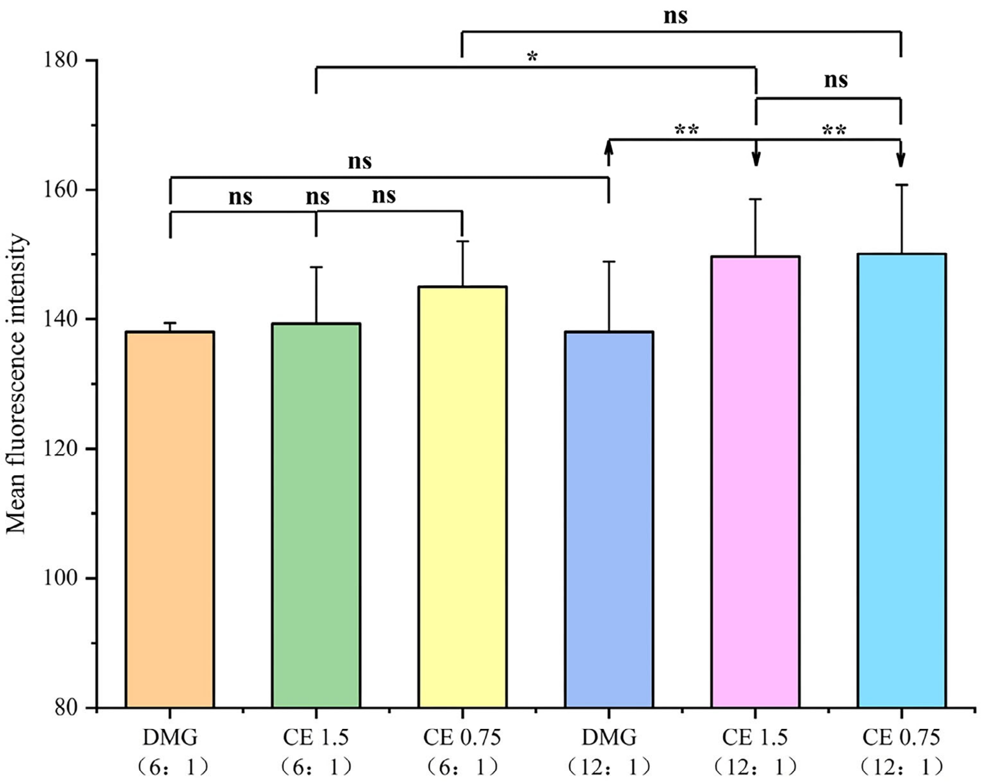

Figure S12. The average fluorescence intensity of DDR1 siRNA LNP ingested at 18 h with different prescriptions and different lipid nucleic acid ratios. ** $P < 0.01$; * $P < 0.05$; ns means $P > 0.05$ ($n = 3$)

Relative Tumor Volume

(1) The relative tumor volume (RTV) of pancreatic cancer tumor-bearing nude mice in different gene siRNA LNP administration groups

The tumor volume (TV) and RTV were calculated for nude mice bearing pancreatic tumors across different siRNA LNP treatment groups. RTV curves for all treated mice are indicated in Figure S13.

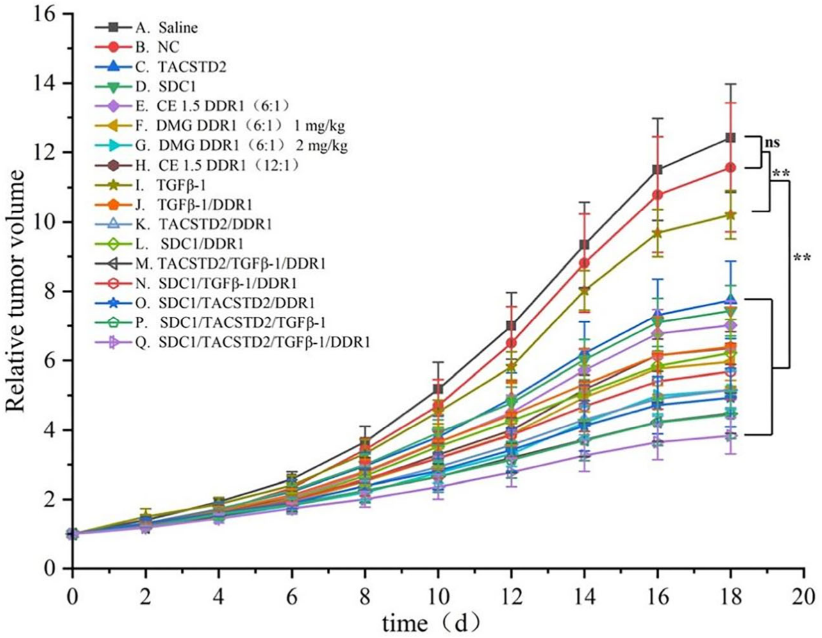

Figure S13. Summary of RTV of cancer-bearing nude mice of different gene siRNA LNP administration groups. $^{**}P < 0.01$; ns means $P > 0.05$ ($n = 7$)

The results revealed that the single siRNA LNP group, the different prescription DDR1 siRNA LNP group, the two siRNA combined LNP group, and the multiple siRNA combined LNP group exhibited significant tumor inhibition effects. The therapeutic effect was significantly better than normal saline and NC siRNA LNP groups; the comparison difference was statistically significant. These results indicated that the four siRNA LNP genes had substantial anti-tumor effects on pancreatic cancer tumor-bearing nude mice.

(a) RTV comparison of pancreatic cancer tumor-bearing nude mice in the single siRNA LNP administration group, the normal saline group, and the NC siRNA LNP group

The RTV mapping curve of cancer-bearing nude mice in the single siRNA LNP administration group is depicted in Figure S14. The inhibitory effect of different siRNA LNP groups on tumor growth was as follows: DDR1 siRNA LNP > SDC1 siRNA LNP > TACSTD2 siRNA LNP > TGFβ-1 siRNA LNP. The efficacies of TGFβ-1 siRNA LNP, TACSTD2 siRNA LNP, SDC1 siRNA LNP, and DDR1 siRNA LNP were significantly better than that of the NC siRNA LNP group. The efficacy of TACSTD2 siRNA LNP, SDC1 siRNA LNP, and DDR1 siRNA LNP was considerably better than that of TGFβ-1 siRNA, and the difference was statistically significant. The efficacy of TACSTD2 siRNA LNP, SDC1 siRNA LNP, and DDR1 siRNA LNP was similar, but the comparison difference was statistically non-significant, and DDR1 siRNA LNP exhibited the best efficacy among single siRNA LNP. The statistical analysis revealed that the single siRNA LNP group had statistically significant differences between normal saline and the NC siRNA LNP groups.

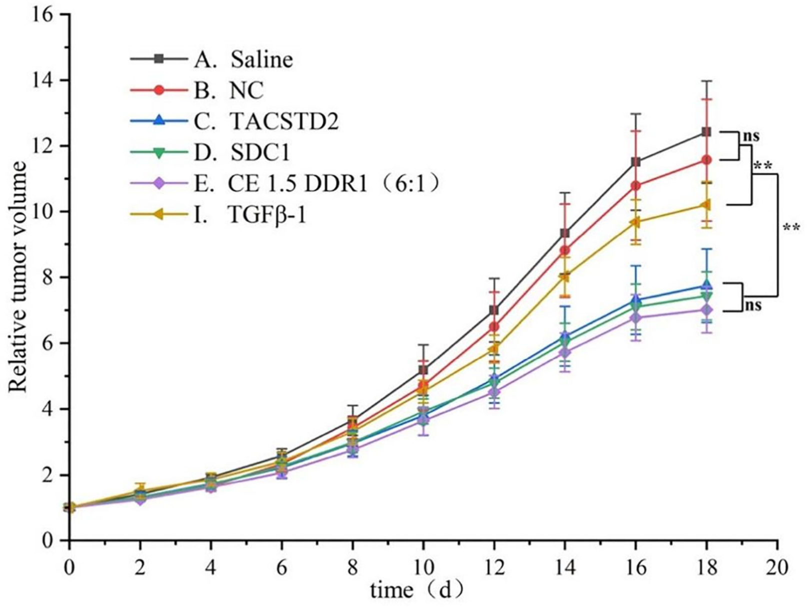

Figure S14. Comparison of RTV in nude mice with pancreatic cancer in the single siRNA LNP administration group. $^{**}P < 0.01$; ns means $P > 0.05$ ($n = 7$)

(b) RTV comparison in nude mice with pancreatic cancer tumors in different prescription DDR1 siRNA LNP administration groups

The RTV mapping curve of pancreatic cancer tumor-bearing nude mice in different DDR1 siRNA LNP administration groups is indicated in Figure S15. The efficacy of CE 1.5 DDR1 siRNA LNP (6:1) 1 mg/kg was slightly less effective than CE 1.5 DDR1 siRNA LNP (12:1) 1 mg/kg, with statistical non-significance. However, the efficacy of CE 1.5 DDR1 siRNA LNP (6:1) 1 mg/kg was significantly less effective than DMG DDR1 siRNA LNP (6:1) at both 1 mg/kg DMG DDR1 siRNA LNP (6:1) and 2 mg/kg. The efficacy of CE 1.5 DDR1 siRNA LNP (12:1) 1 mg/kg was slightly weaker than DMG DDR1 siRNA LNP (6:1) 1 mg/kg, and the difference was statistically non-significant. The efficacy of DMG DDR1 siRNA LNP (6:1) 1 mg/kg was weaker than that of DMG DDR1 siRNA LNP (6:1) 2 mg/kg, and the difference was statistically significant.

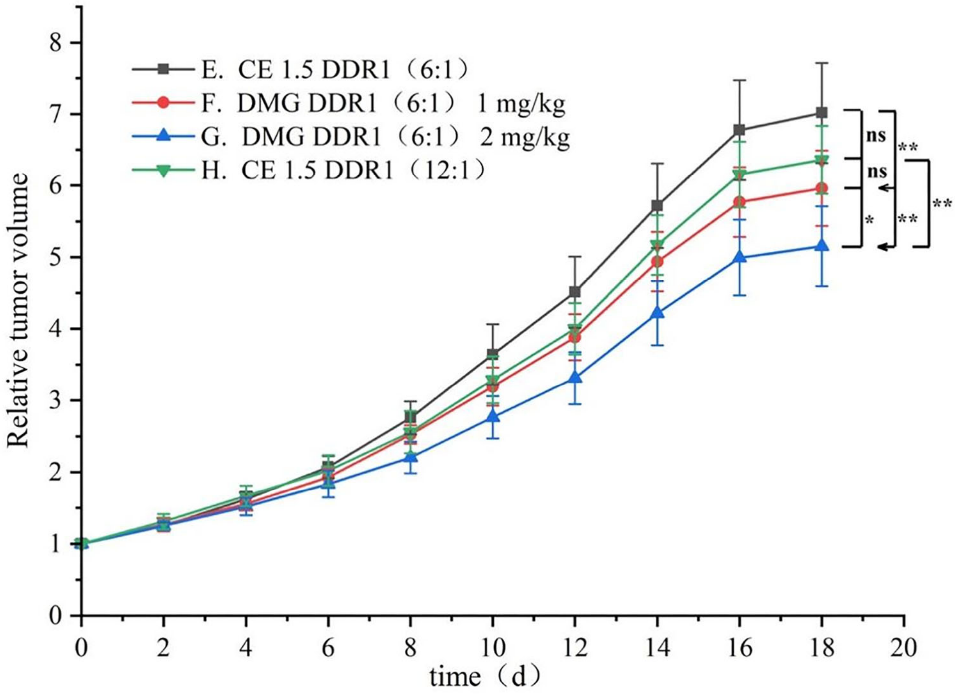

Figure S15. Comparison of RTV in nude mice with pancreatic cancer tumors in different DDR1 siRNA LNP administration groups. $^{**}P < 0.01$; $^{*}P < 0.05$; ns means $P > 0.05$ ($n = 7$)

(c) RTV comparison in nude mice with pancreatic cancer in single and double siRNA LNP administration groups

The RTV mapping curve of nude mice with a pancreatic cancer tumor in the single siRNA and double siRNA LNP administration group is depicted in Figure S16. TGFβ-1/DDR1 siRNA LNP was slightly stronger than DDR1 siRNA LNP, and the difference was statistically non-significant. SDC1/DDR1 siRNA LNP was marginally more potent than DDR1 siRNA LNP, and the difference was statistically non-significant. Moreover, SDC1/DDR1 siRNA LNP was stronger than SDC1 siRNA LNP, and the difference was statistically significant. The efficacy of TACSTD2/DDR1 siRNA LNP was significantly better than TACSTD2 siRNA LNP and DDR1 siRNA LNP; the comparison difference was statistically significant, and the combined effect was noticeable.

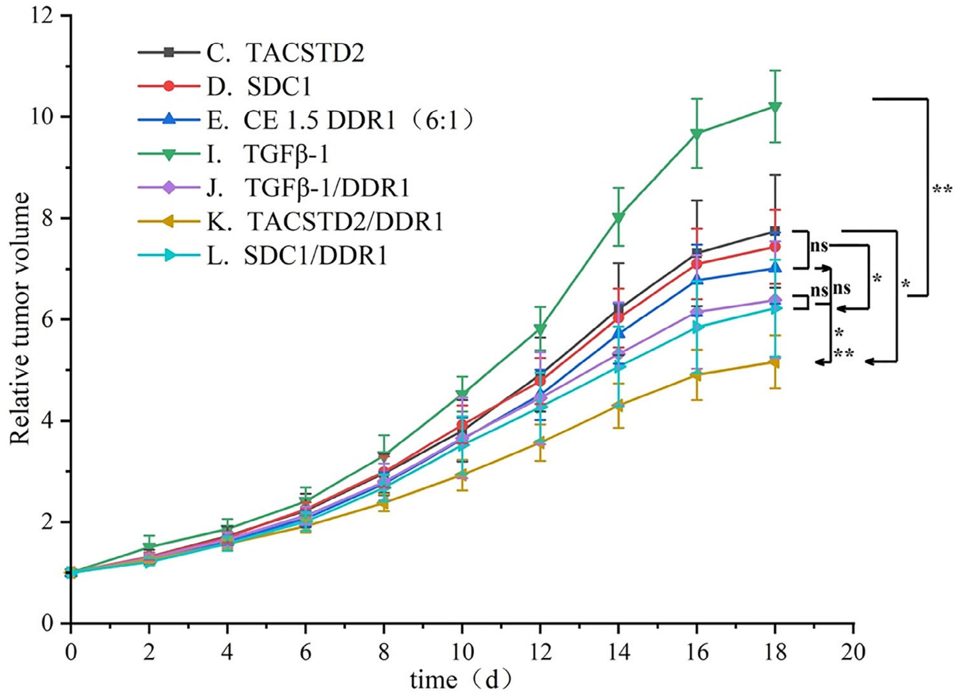

Figure S16. Comparison of RTV in nude mice with pancreatic cancer in the single and double siRNA LNP administration groups. ** $P < 0.01$; * $P < 0.05$; ns means $P > 0.05$ ($n = 7$)

(d) RTV comparison in nude mice with a pancreatic cancer tumor in the multiple siRNA combined with LNP administration group

The RTV mapping curve of pancreatic cancer tumor-bearing nude mice in the multiple siRNA combined LNP administration group is depicted in Figure S17. The efficacy of SDC1/TACSTD2/TGFβ-1 siRNA LNP was comparable to that of TACSTD2/TGFβ-1/DDR1 siRNA LNP, with statistical non-significance. The efficacy of TACSTD2/TGFβ-1/DDR1 siRNA LNP was superior to that of TACSTD2/DDR1 siRNA LNP and TGFβ-1/DDR1 siRNA LNP, and the combined effect was apparent, and the comparative difference was statistically significant. The combined impact of SDC1/TACSTD2/TGFβ-1/DDR1 siRNA LNP was significantly better than TGFβ-1/DDR1 siRNA LNP, SDC1/DDR1 siRNA LNP, and TACSTD2/DDR1 siRNA LNP, and the difference was statistically significant. The combined effect of SDC1/TACSTD2/TGFβ-1/DDR1 siRNA LNP was better than that of SDC1/TACSTD2/DDR1 siRNA LNP, and the difference was statistically significant. The combined effect of SDC1/TACSTD2/TGFβ-1/DDR1 siRNA LNP was better than that of TACSTD2/TGFβ-1/DDR1 siRNA LNP, and the difference was statistically non-significant.

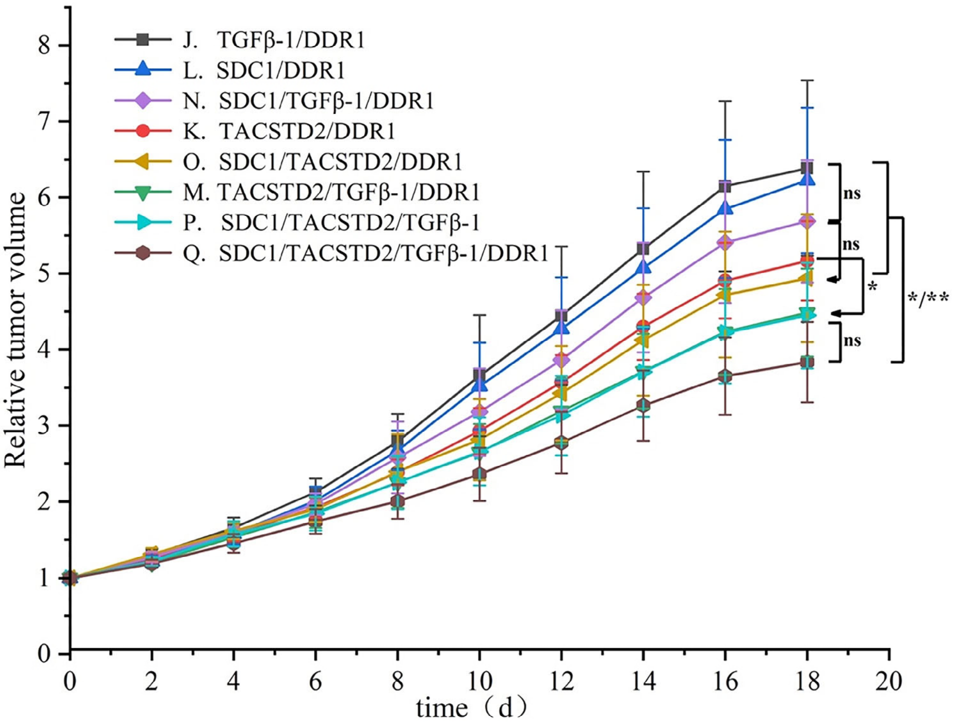

Figure S17. Comparison of RTV in nude mice with pancreatic cancer tumors in the multiple siRNA combined with LNP administration group. ** $P < 0.01$; * $P < 0.05$; ns means $P > 0.05$ ($n = 7$)

(e) RTV comparison between the three- and four-siRNA combined with LNP administration group (total dose: 1 mg/kg) and the DMG DDR1 siRNA LNP administration group (2 mg/kg) in nude mice with pancreatic cancer

The RTV plot curve of pancreatic cancer tumor-bearing nude mice treated with either three- or four-siRNA combined LNP administration groups (total dose: 1 mg/kg) and DMG DDR1 siRNA LNP 2 mg/kg administration group is indicated in Figure S18. RTV comparison between the three and four siRNA combined LNP groups and the DMG DDR1 siRNA LNP 2 mg/kg demonstrated that the efficacy of SDC1/TACSTD2/TGFβ-1 siRNA LNP and TACSTD2/TGFβ-1/DDR1 siRNA LNP was comparable, with a statistically non-significant difference. Similarly, the therapeutic efficacy of the three-siRNA LNP combinations—TACSTD2/TGFβ-1/DDR1 siRNA LNP, SDC1/TGFβ-1/DDR1 siRNA LNP, SDC1/TACSTD2/DDR1 siRNA LNP, and SDC1/TACSTD2/TGFβ-1 siRNA—all at a total dose of 1 mg/kg, was similar to that of DMG DDR1 siRNA LNP 2 mg/kg, with statistical non-significance. These findings suggest that the efficacy of three kinds of siRNA combined with LNP was similar to that of doubling the dose of a single siRNA LNP, and the combined effect was significant. The efficacy of SDC1/TACSTD2/TGFβ-1/DDR1 siRNA LNP was significantly better than that of DMG DDR1 siRNA LNP 2 mg/kg, and the difference was statistically significant, indicating that multiple siRNA combined with LNP could significantly improve efficacy and even be better than doubling the dose of a single siRNA LNP.

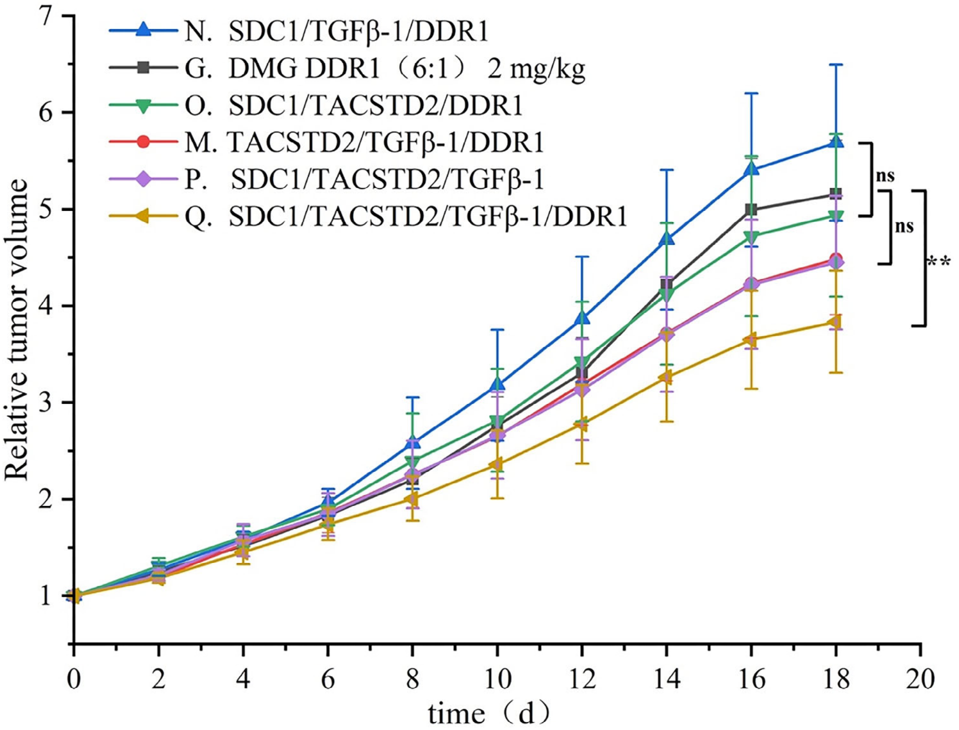

Figure S18. Comparison of RTV in nude mice with pancreatic cancer tumors in the three and four siRNA combined LNP administration group (total dose of 1 mg/kg) and DMG DDR1 siRNA LNP administration group (2 mg/kg). ** $P < 0.01$; ns means $P > 0.05$ ($n = 7$)

(2) The relative tumor proliferation rate of pancreatic cancer tumor-bearing nude mice in different gene siRNA LNP administration groups

Based on the TV of pancreatic cancer-bearing nude mice detected during administration, the relative tumor proliferation rate was calculated, and the relative tumor proliferation rate curve of LNP administration groups with different gene siRNA was concluded, as depicted in Figure S19. The results of statistical analysis revealed that the therapeutic effect of the single siRNA LNP group, DDR1 siRNA LNP group with different prescriptions, and multiple siRNA combined LNP group were significantly superior to the normal saline group and NC siRNA LNP group, with a significant inhibitory effect on tumor growth, and the comparison difference was statistically significant.

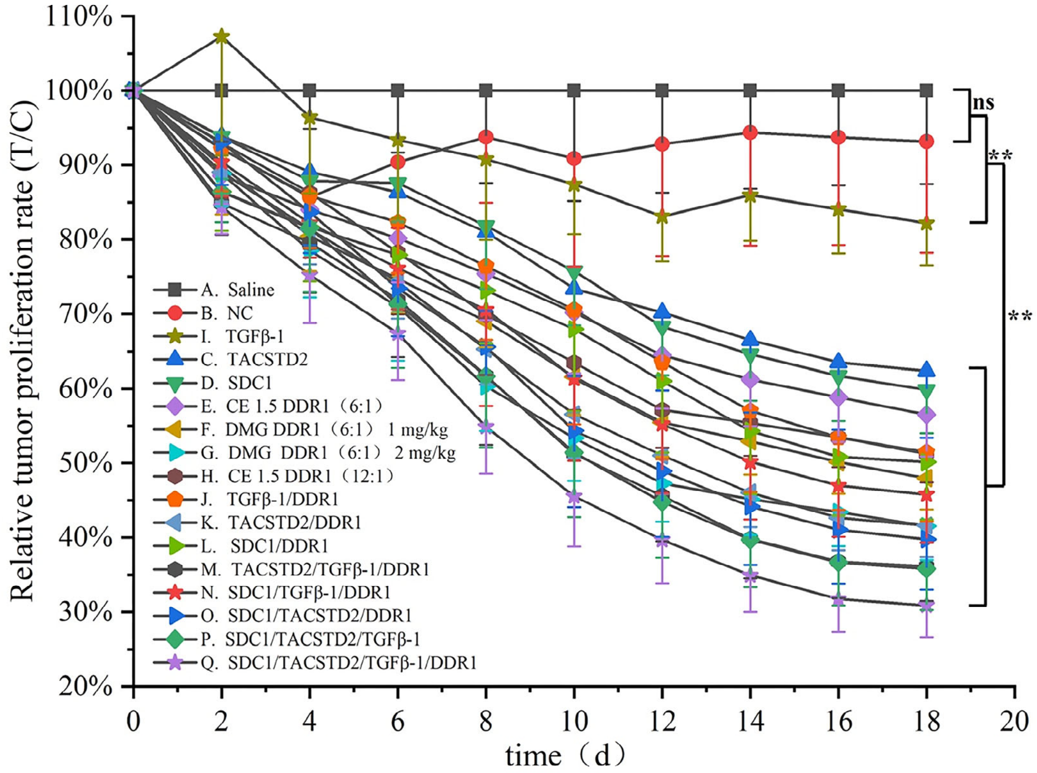

Figure S19. T/C summary of pancreatic cancer tumor-bearing nude mice in different gene siRNA LNP administration groups. ** $P < 0.01$; ns means $P > 0.05$ ($n = 7$)

The normal saline group and the NC siRNA LNP group exhibited no therapeutic effect, while the therapeutic effect of the other siRNA LNP groups was significantly better than that of the NC siRNA LNP group and the normal saline group. The statistical results of each group were consistent with those of RTV. In conclusion, all target gene siRNA LNP administration groups were effective. Among the single siRNA LNP comparisons, TACSTD2 siRNA LNP, SDC1 siRNA LNP, and DDR1 siRNA LNP were superior to the TGFβ-1 siRNA LNP group, and the difference was statistically significant. The efficacies of TACSTD2 siRNA LNP, SDC1 siRNA LNP, and DDR1 siRNA LNP were similar, and the comparison difference was statistically non-significant. The efficacy of increasing the lipid nucleic acid ratio was slightly increased, but it could not reach the extent of improving the effectiveness of multiple siRNA combined with LNP. Dose-doubling increased the efficacy slightly but non-significantly. However, the efficacy of several siRNA combined with the LNP group (no dose increase) was equivalent to that of a single siRNA LNP group. Some siRNA combined with the LNP group (no dose increase) was much stronger than that of the single siRNA LNP group, and the combined effect of some siRNA combined with the LNP group was pronounced. Among them, SDC1/TACSTD2/TGFβ-1/DDR1 siRNA LNP, TACSTD2/TGFβ-1/DDR1 siRNA LNP, and SDC1/TACSTD2/TGFβ-1 siRNA LNP had the most significant improvement in efficacy.

(3) The growth and weight changes of nude mice with pancreatic cancer tumors in different siRNA LNP administration groups

According to the weight of tumor-bearing nude mice of pancreatic cancer measured during administration, the growth and weight change curves of tumor-bearing nude mice of different

administration groups with siRNA LNPs were drawn, as depicted in Figure S20. During treatment, tumor-bearing nude mice of pancreatic cancer in all administration groups were in good condition, with non-significant weight loss and no death compared with the initial weight. Compared with the synchronous saline group, the weight of each treatment group, especially the two, three, and four siRNA combined LNP group, decreased slightly in the later stage of treatment, indicating that the combination of multiple siRNA with LNP was slightly toxic, and the pros and cons of efficacy and toxicity should be measured. However, as pancreatic cancer is challenging to treat and has a high fatality rate, slight drug toxicity is usually acceptable.

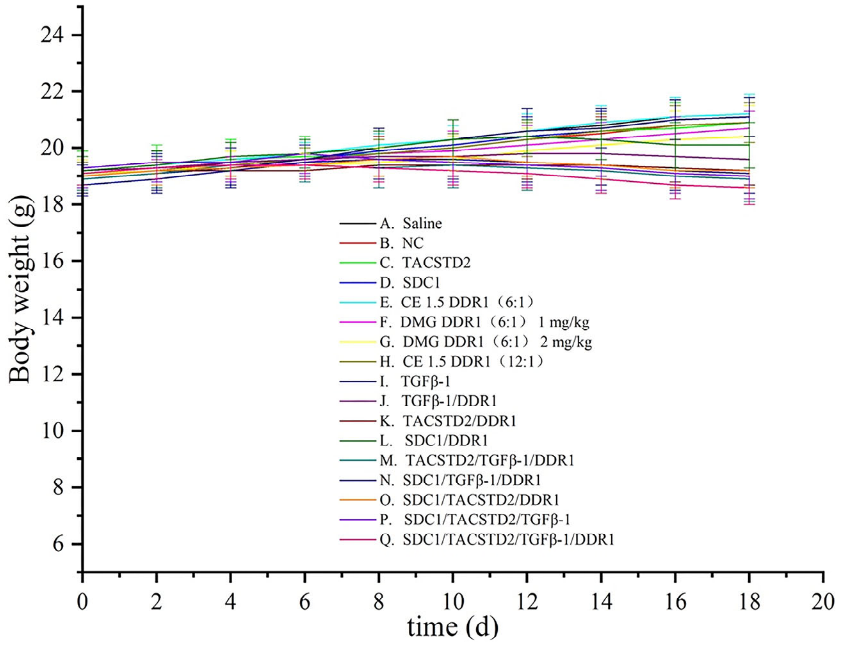

Figure S20. Effects of LNP administration groups with different siRNA genes on body weight of nude mice bearing pancreatic cancer (n = 7)
